# Transportin 3 Promotes a Nuclear Maturation Step Required for Efficient HIV-1 Integration

**DOI:** 10.1371/journal.ppat.1002194

**Published:** 2011-08-25

**Authors:** Lihong Zhou, Elena Sokolskaja, Clare Jolly, William James, Sally A. Cowley, Ariberto Fassati

**Affiliations:** 1 Wohl Virion Centre, Division of Infection & Immunity, University College London, London, United Kingdom; 2 MRC Centre for Medical Molecular Virology, Division of Infection & Immunity, University College London, London, United Kingdom; 3 Sir William Dunn School of Pathology, University of Oxford, Oxford, United Kingdom; Fred Hutchinson Cancer Research Center, United States of America

## Abstract

The HIV/AIDS pandemic is a major global health threat and understanding the detailed molecular mechanisms of HIV replication is critical for the development of novel therapeutics. To replicate, HIV-1 must access the nucleus of infected cells and integrate into host chromosomes, however little is known about the events occurring post-nuclear entry but before integration. Here we show that the karyopherin Transportin 3 (Tnp3) promotes HIV-1 integration in different cell types. Furthermore Tnp3 binds the viral capsid proteins and tRNAs incorporated into viral particles. Interaction between Tnp3, capsid and tRNAs is stronger in the presence of RanGTP, consistent with the possibility that Tnp3 is an export factor for these substrates. In agreement with this interpretation, we found that Tnp3 exports from the nuclei viral tRNAs in a RanGTP-dependent way. Tnp3 also binds and exports from the nuclei some species of cellular tRNAs with a defective 3′CCA end. Depletion of Tnp3 results in a re-distribution of HIV-1 capsid proteins between nucleus and cytoplasm however HIV-1 bearing the N74D mutation in capsid, which is insensitive to Tnp3 depletion, does not show nucleocytoplasmic redistribution of capsid proteins. We propose that Tnp3 promotes HIV-1 infection by displacing any capsid and tRNA that remain bound to the pre-integration complex after nuclear entry to facilitate integration. The results also provide evidence for a novel tRNA nucleocytoplasmic trafficking pathway in human cells.

## Author Summary

HIV-1, the causative agent of AIDS, is a virus that enters the nucleus of infected cells and must integrate its genome into the host cell DNA. Here we show that efficient HIV-1 integration depends on a host cell factor called Transportin 3. We also show that Transportin 3 can export out of the nucleus the viral capsid proteins and viral transfer RNAs (tRNAs) and that this activity is important for HIV-1 integration. We propose that Transportin 3 facilitates a maturation process inside the nucleus by removing remaining capsid proteins and tRNAs still bound to the virus. The mature viral complex, free of any bulky component, can then more easily integrate. Transportin 3 also export certain cellular tRNAs out of the nucleus, presumably as a way to control cell metabolism. By feeding into this tRNA shuttling pathway, HIV-1 can complete its life cycle. Our work sheds new light into the biology of HIV-1 and points to the existence of a new pathway in human cells to shuttle certain tRNAs between nucleus and cytoplasm.

## Introduction

Akin to other viruses [1], after cell-receptor mediated entry into the cell, HIV-1 undergoes an uncoating step by shedding its capsid core [2], [3], which is constituted by approximately 1,500 capsid proteins (CA) arranged in a hexameric lattice [4]. This step is incompletely understood yet it is important to maintain optimal infectivity [3], [5], [6]. If uncoating of the viral core takes place too early, infectivity is impaired as observed with viral mutants having unstable capsid cores or in the presence of certain members of the TRIM protein family [5], [7], [8]. If the virus uncoats too late or incompletely, infectivity is also impaired [5], [9]. Interestingly, proper uncoating of the viral core, which is thought to take place in the cytoplasm of infected cells during reverse transcription [6], [10], can also influence later events such as nuclear entry and integration [9], [11].

Changes in CA have been shown to impact on HIV-1 nuclear import and infection of non-dividing cells in several ways. Substitution of HIV-1 CA with MLV CA impairs HIV-1 ability to infect non-dividing cells [12]. This CA substitution makes HIV-1 phenotypically similar to the murine leukemia virus (MLV), which cannot efficiently infect non-dividing cells and maintains relatively large amounts of CA associated with its reverse transcription complex (RTC) [13]. HIV-1 CA may determine which components of the nuclear pore complex (NPC) are preferentially used for infection, because specific mutations in CA make the virus less dependent on NUP153 and more dependent on NUP155 [14]. Furthermore, CA influences incorporation of certain tRNAs species into the viral particle, which promote HIV-1 entry into the nucleus, presumably by recruiting the intracellular viral complex into the so called tRNA retrograde transport pathway [15], [16].

CA also impacts on post-nuclear entry events. HIV-1 mutants that maintain larger amounts of CA associated with their RTCs and pre-integration complexes (PICs) integrate less efficiently [9]. Furthermore, the restriction factors TRIMcyp and TRIM19, which bind to CA, can block post-nuclear entry steps required for efficient integration [17]. This evidence supports a functional link between CA, uncoating, nuclear import and integration [9], [11], [17], however a unifying picture is lacking and little is known of the events taking place between HIV-1 nuclear entry and integration.

Interestingly, recent high throughput screenings showed that Transportin 3 (Tnp3) (Gene ID: 23534) is a host factor critical for some events occurring at or shortly after HIV-1 nuclear import [18], [19], [20], [21]. Tnp3 belongs to the importin ß superfamily of nuclear transport receptors that bind RanGTP at their N-termini [22] and participate in nucleocytoplasmic transport of proteins and nucleic acids by interacting with specific FG-repeats present in many nucleoporins, the constituents of the NPC [23].

Members of the importin ß superfamily can act as nuclear import or export receptors (or both) depending on whether they bind or release the cargo in the presence of RanGTP. Nuclear *import* receptors bind their cargos in the cytoplasm and release them in the nucleus upon binding to RanGTP, whereas nuclear *export* receptors bind their cargos in the nucleus in complex with RanGTP and dissociate from them in the cytoplasm upon hydrolysis of RanGTP [24]. In the nucleus, RanGDP in enzymatically converted into RanGTP by the regulator of chromatin condensation-1 (RCC1), a chromatin-bound guanine nucleotide exchange factor, whereas on the cytoplasmic face of the NPC RanGTP is hydrolyzed into RanGDP by the Ran GTPase-activating protein (RanGAP1) and RanBP1 [24]. Hence a concentration gradient of RanGTP (high in the nucleus and low in the cytoplasm) is maintained across th nuclear envelope, providing directionality to nucleocytoplasmic trafficking.

We have investigated the role of Tnp3 in HIV-1 infection and found that it is important for the completion of a post nuclear-entry step. We show that Tnp3 binds to CA and tRNA species present in the viral particle in a RanGTP-dependent way, facilitates their nuclear export and may promote a maturation step of the PIC inside the nucleus required for efficient integration. Remarkably, we found that Tnp3 is also an export factor for certain cellular tRNA species lacking a complete 3′ CCA end.

## Results

### Tnp3 is required for efficient HIV-1 integration

**Figure 1:**
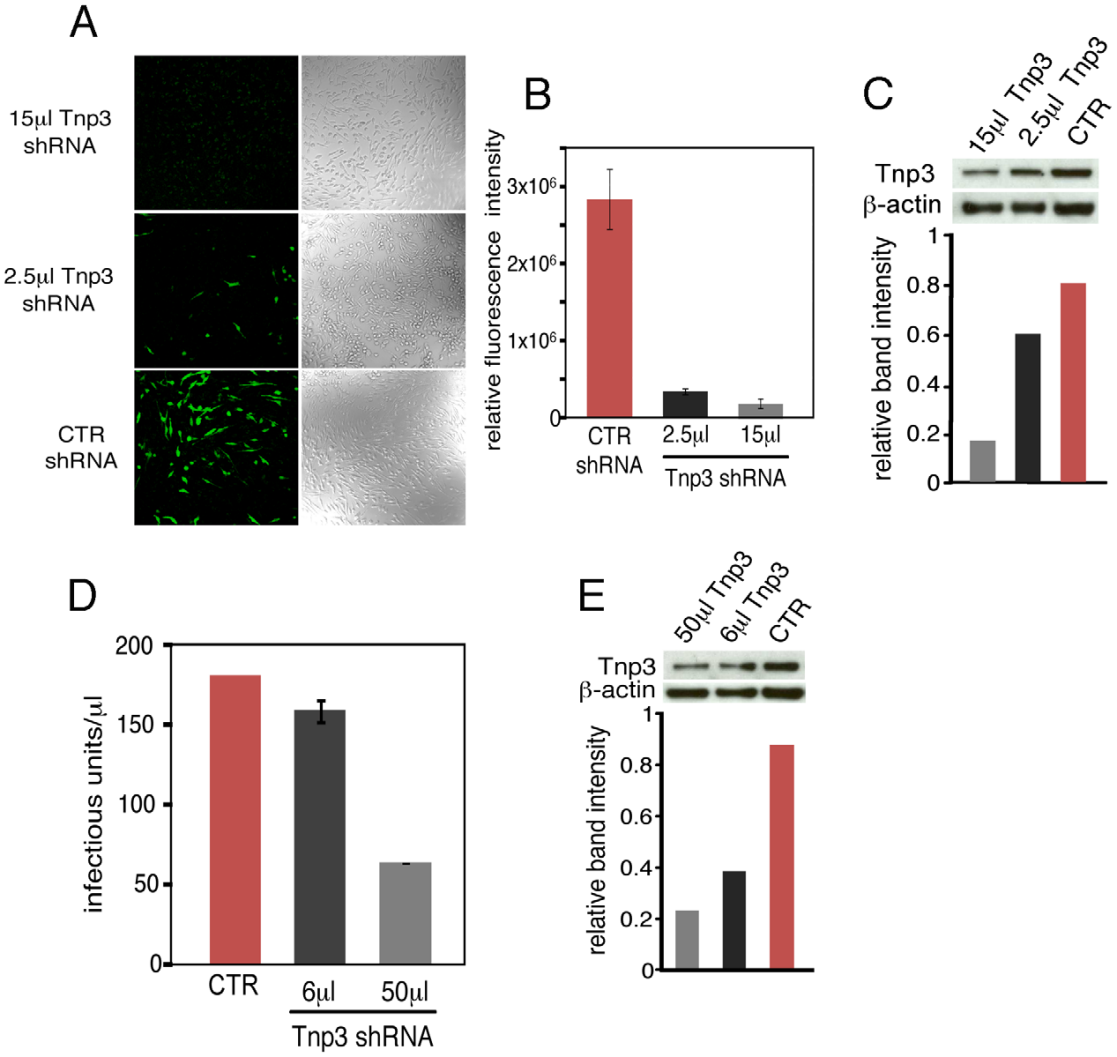
Tnp3 supports HIV-1 infection in human macrophages. (A) HuES-2 derived macrophages were transduced with two different doses of a lentivirus vector delivering an shRNA against human Tnp3 mRNA, or a control shRNA against dsRed mRNA and infected with a fixed amount of HIV_GFP_ vector 96 hours later. Five days after infection with the HIV_GFP_ vector cells were visualized by confocal microscopy. (B) ImageJ software was used to calculate the fluorescence intensity per cell relative to un-infected cells (zero value). At least 100 cells per field were counted. Average ± SD of triplicate experiments are shown. (C) Knockdown of Tnp3 was demonstrated by Western blotting (upper panel). The degree of Tnp3 knockdown was calculated by determining intensities of Tnp3 bands relative to actin bands using ImageJ software (lower panel). (D) A fixed amount of HIV_GFP_ vector was used to infect blood-derived macrophages previously transduced with the shRNA lentiviral vector as described in (A). GFP-positive cells were visualised using confocal microscopy and infectious units calculated by counting the number of GFP+ cells per well. Average values ± SD of triplicate experiments are shown. (E) Knockdown of Tnp3 was demonstrated in blood-derived macrophages by Western blotting (upper panel). The degree of Tnp3 knockdown was calculated by determining intensities of Western blot bands using ImageJ software (lower panel).

**Figure 2:**
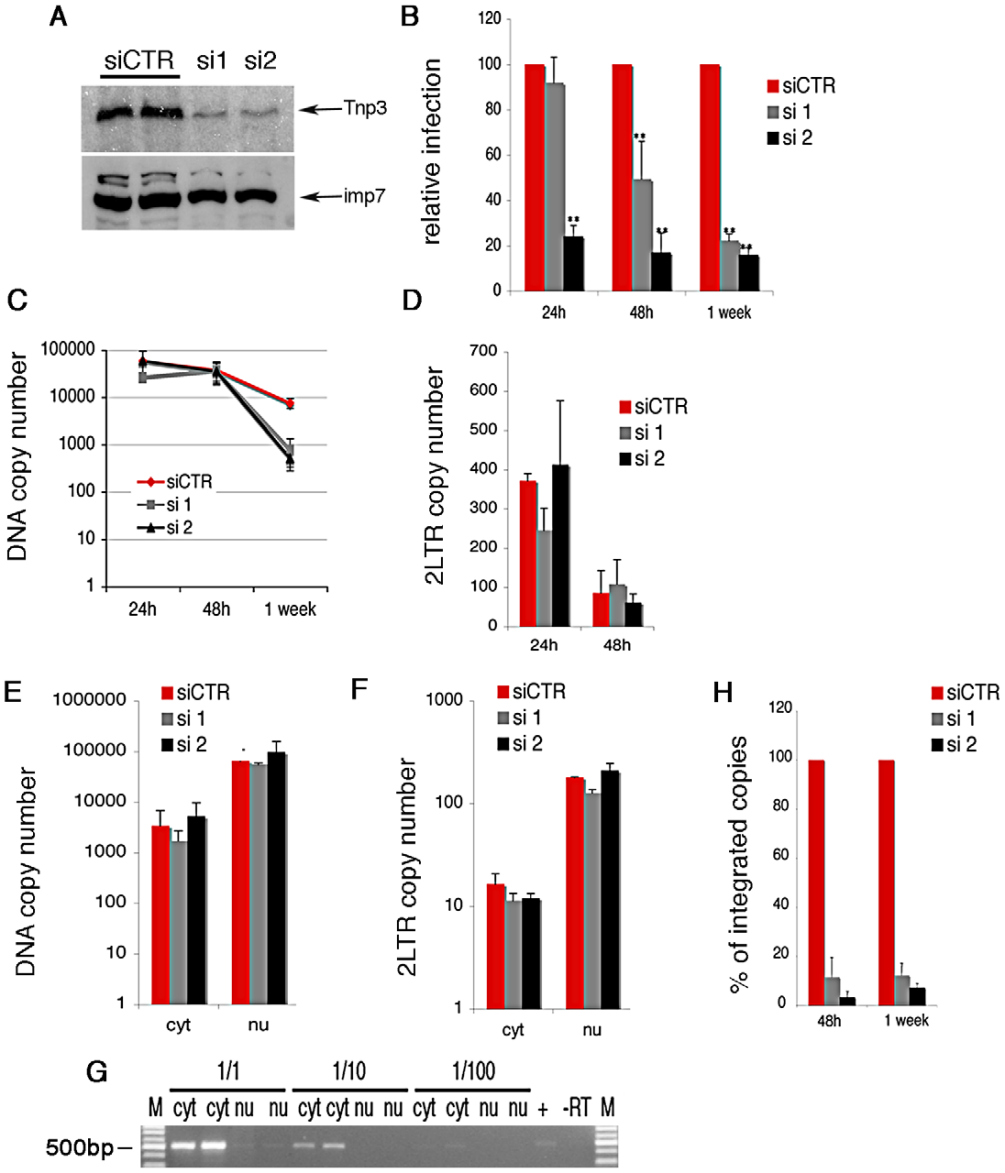
Tnp3 is required for efficient HIV-1 infection in HeLa cells. (A) Cells were transfected with a scramble siRNA or two different siRNAs targeting Tnp3 mRNA and analyzed by Western blot 72 h later; importin 7 (imp7) is used as a loading control. (B) Cells were transduced with HIV-1_GFP_ vector 48h after siRNA transfection and the number of infected cells measured by flow cytometry at the indicated time points post-infection. Average values ± SD of three independent experiments are shown (** p<0.01 Student's t-test). (C) Reverse transcription and (D) accumulation of 2LTRs circular DNA forms in Tnp3 KD cells were measured by Taqman qPCR. (E-F) Infected cells were fractionated into a cytoplasmic (cyt) and nuclear (nu) fraction 24 h post-infection. Total viral DNA (E) and 2LTRs viral DNA forms (F) were quantified by TaqMan qPCR in each fraction. (G) RNA was extracted from equal volumes of each fraction and reverse transcribed into cDNA. The spliced cyclophilin A mRNA was detected by PCR (500 bp) to control for cross contamination of the fractions. M, DNA molecular weight markers. (H) DNA extracted from infected cells was subjected to Alu-LTR TaqMan qPCR to detect integrated HIV-1 copies. Signal from the 24 h time point could not be detected consistently. Average values ± mean variation from two independent experiments analyzed in triplicate are shown.

We wanted to investigate the role of Tnp3 in cell types relevant to HIV-1 infection, including macrophages and CD4+ T-cells. To this end, Tnp3 was depleted in human embryonic stem (ES) cell-derived macrophages [25] by lentiviral delivery of an shRNA targeting Tnp3 mRNA or the DsRed mRNA as control [26] and cells were infected four days later with an HIV-1 vector bearing the green fluorescent protein expression cassette (HIV_GFP_). Levels of Tnp3 appeared to be low in differentiated macrophages and even a fairly modest depletion of Tnp3 resulted in ∼10-fold inhibition of HIV-1_GFP_ infection (Figure 1A-C). A greater depletion of Tnp3 corresponded to a greater block to HIV_GFP_ infection (Figure 1A-C). Similar results were obtained in blood-derived macrophages (Figure 1D, 1E), in agreement with Christ et al. [20]. Next, we depleted Tnp3 in CD4+ Jurkat T-cells using a MLV vector to deliver an shRNA targeting Tnp3 mRNA. Relative to macrophages, HIV-1_GFP_ infection was modestly (∼3 fold) but consistently inhibited in Jurkat Tnp3 knock-down (KD) cells, despite effective Tnp3 depletion (Figures S1A, S1B). We also depleted Tnp3 in HeLa cells by using two different siRNAs. Depletion of Tnp3 in HeLa cells resulted in ∼7 fold inhibition of infection with the best siRNA (Figure 2A, 2B) in agreement with previous reports [18], [20]. Hence Tnp3 supports efficient HIV-1 infection in relevant target cells although different cell types might require different levels of Tnp3 to that end.

We used HeLa cells, which can be grown in sufficiently large quantity and showed a robust phenotype, to examine in more detail which step of the HIV-1 life cycle was affected by depletion of Tnp3 (Figure 2). Reverse transcription of the viral RNA genome was not impaired in HeLa Tnp3 KD cells, however we detected 10 fold less viral DNA one week post-infection (Figure 2C), suggesting that viral nuclear import or integration (or both) were defective. To investigate viral nuclear entry, levels of 2LTRs circular DNA (a viral DNA form that is generated by end-ligation inside the nucleus) [27] were measured by TaqMan qPCR at 24 h and 48 h post-infection. Similar amounts of 2LTR circular DNA were detected in control and KD cells (Figure 2D). Infected cells were also fractionated and the distribution of viral DNA in the cytoplasm and nucleus was examined by TaqMan qPCR. A similar distribution of viral DNA in the nuclear and cytoplasmic fractions of Tnp3 KD and control cells suggested that viral nuclear import was not defective (Figure 2E). The quality of the fractionation was controlled two fold. First, 2LTR circular viral DNA was enriched >20 fold in the nuclear fractions, consistent with the notion that viral DNA is circularized inside the nucleus [27] (Figure 2F). Second, the distribution of spliced cyclophilin A mRNA in each fraction showed that contamination of nuclei with cytoplasmic material was <5% [26] (Figure 2G). This result also indicated that fractionated nuclei were mostly devoid of the external nuclear envelope layer, which is contiguous with the ER. To test if viral DNA could be bound to the nuclear envelope, we fractionated infected cells in the presence of DNAse I beads. Whereas DNAse beads could digest in part viral DNA in the cytoplasm, viral DNA in the nuclear fraction was unaffected, suggesting that it was inside the nuclei and protected from digestion (Figure S2). Since viral nuclear import was not significantly inhibited, we next measured integration by Alu-PCR Taqman qPCR. We found that the amount of integrated viral DNA was approximately 10 fold lower in Tnp3 KD cells compared to control cells [28], [29] (Figure 2H). Similar data were obtained with a near full length HIV-1 clone containing all accessory proteins (HIV-1_LAI_Denv) [12] (Figure S3). Although the block to HIV-1_GFP_ infection was modest in Jurkat Tnp3 KD cells, we found that integration was the only step significantly inhibited in these cells, consistently with the results obtained in HeLa cells (Figure S1). These results showed that Tnp3 is required for a post-nuclear entry step leading to efficient integration.

### Tnp3 binds to CA and viral tRNAs

Tnp3 was shown to bind to HIV-1 integrase (IN), but the biological relevance of this interaction is uncertain [19], [20]. To investigate if Tnp3 bound to other viral elements in addition to IN and to gain a greater understanding of its function, purified virus was used in pull down assays with recombinant Tnp3 in the presence or absence of the RanQ69L-GTP mutant. The RanQ69L point mutant is hydrolyzed to the GDP form with a kinetic several orders of magnitude slower than wild type RanGTP [30], making it more suitable for pull down assays. Addition of RanGTP to the *in vitro* binding assays is critical to understand if the nuclear transport receptor acts as an import or an export factor for the specific cargo and adds an important element of specificity to the assay.

**Figure 3:**
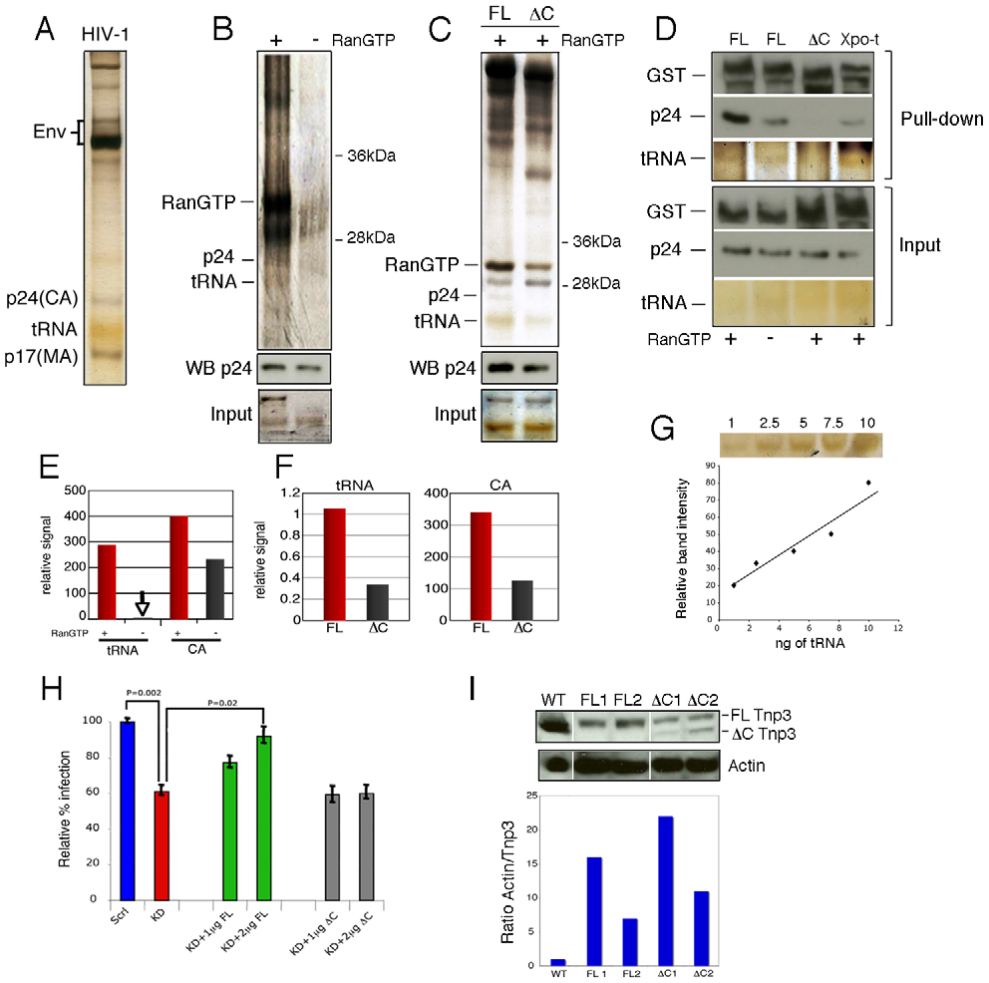
Tnp3 binds viral tRNA and CA. (A) Purified HIV-1 vector used for pull down assays was visualized by silver staining after 15% denaturing PAGE. Env, viral envelope glycoproteins; p24(CA), capsid proteins; p17(MA), matrix proteins. (B) Pull down assay with purified virus and GST-Tnp3 in the presence or absence of RanQ69L-GTP. (C) Pull down assay with purified virus and GST-Tnp3 (FL) or GST-Tnp3 DC825 (ΔC) deletion mutant in the presence of RanQ69L-GTP. Unlabelled bands are impurities from the Tnp3 preparations. WB p24, Western blot with an anti-capsid antibody. (D) Pull down assay with GST-Tnp3 (FL), GST-Tnp3 DC825 (ΔC) or GST-Xpo-t (Xpo-t). Samples were analyzed by silver stain and by Western blot with anti-capsid and anti-GST antibodies. (E) ImageJ quantification of tRNA and CA in pull down assays as shown in B. RanGTP increased CA binding to Tnp3 by 2.5±0.5 fold in three independent experiments. (F) ImageJ quantification of pull down assays as shown in C. Values are expressed as ratio of input versus recovered tRNAs. DC825 Tnp3 had 3±0.5 fold reduced binding to CA in two independent experiments. (G) tRNA titration curve after silver staining of the SDS-PAGE gel. (H) Rescue experiment. Polyclonal populations of scramble (Scrl) or Tnp3 KD (KD) 293T cells were transfected with plasmid DNA expressing full length (FL) Tnp3 or Tnp3 DC825 bearing a silent point mutation to make them resistant to the shRNA effect (ΔC). Cells were transduced with an HIV-1_GFP_ vector and analyzed by flow cytometry 24 hours later. Mean values ± SD of four independent experiments are shown; Student's t-test was used to calculate statistical significance. (I) Western blot to detect Tnp3 expression in transfected cells. WT, Scrl cells; FL1 and FL2, KD cells transfected with 1 µg and 2 µg Tnp3 cDNA respectively; ΔC1 and ΔC2, KD cells transfected with the same amounts of Tnp3 DC825 cDNA. Bottom panel, quantification of the Tnp3 signal. To quantify signal in FL samples, the background from pre-existing Tnp3 (as detected in the DC825 cDNA-transfected samples) was subtracted and all values were normalized for actin input.

Viral stocks were prepared from stable HIV-1 vector producer cells pseudotyped with the amphotropic envelope [31], purified through two sucrose gradients and analyzed by SDS-PAGE and silver staining. This procedure allowed visualization of several viral proteins including Env, capsid (CA) and matrix (MA). tRNAs incorporated into viral particles [32] were also clearly visible as the typical yellowish band migrating at ∼20 kDa (Figure 3A). Pull-down assays were performed with virus particles mildly disrupted by gentle sonication in the absence of detergents to limit the damage to viral cores. Viral tRNAs and CA bound to Tnp3 with greater affinity in the presence of RanGTP (Figure 3B), implying that Tnp3 might be an export factor for these molecules [33]. Next we generated several Tnp3 deletion mutants (see below) and found that a mutant lacking the last C-terminal 98 residues bound both viral tRNAs and CA less well, suggesting that these viral components may recognize the same Tnp3 domain (Figure 3C). To confirm the specificity of the interaction between Tnp3 and CA, we performed parallel pull-down assays with wild type Tnp3 with or without RanGTP, the C-term deleted Tnp3 and Exportin-t (Figure 3D). Exportin-t (Xpo-t) is the main tRNA export receptor in mammalian cells and binds tRNAs with high affinity in the presence of RanGTP [34], [35], [36], [37]. We confirmed that Tnp3 binds CA and tRNAs with higher affinity in the presence of RanGTP and that the C-term deleted Tnp3 did not bind efficiently to either. Importantly, Xpo-t did bind to viral tRNAs, consistent with the notion that viral particles contain a mix of normal and truncated tRNAs [15], but did not bind efficiently to HIV-1 CA (Figure 3D). The relevant tRNAs and CA gel bands were quantified using ImageJ software analysis tool, which confirmed the trend observed by visual inspection (Figure 3E-G). Hence, the interaction between Tnp3 and CA is specific. Interestingly, we were unable to detect a significant interaction between Tnp3 and purified recombinant CA, similarly to Fv1, TRIM5α and CPSF6 [14].

To test the biological significance of the pull down assays and the importance of the last 98 C-term residues of Tnp3, we performed rescue experiments. A polyclonal population of 293T cells with a stable Tnp3 KD was generated, which showed a modest but highly reproducible defect for HIV-1 infection (Figure 3H). The Tnp3 KD cells were then transfected with increasing doses of a plasmid expressing wild type or the C-term truncated Tnp3 with a point mutation to make the mRNAs resistant to the shRNA targeting. Cells were challenged with the HIV-1_GFP_ vector 48h after transfection and then analyzed by flow cytometry. Transfection of the plasmid expressing full length Tnp3 partially rescued HIV-1 vector infection; in contrast, the plasmid expressing the C-term deleted Tnp3 showed no rescue phenotype at all (Figure 3H). Tnp3 levels were monitored by Western blotting: 293T cells showed a substantial KD and even a modest recovery of Tnp3 expression above background levels was sufficient for a partial rescue of HIV-1 vector infection (Figure 3I). In contrast, similar levels of expression of the C-term deleted Tnp3 did not have any effect on HIV-1_GFP_ transduction (Figure 3I). Hence, the last 98 residues of Tnp3 are important for CA, tRNA binding and HIV-1_GFP_ infection.

### Tnp3 binds to cellular tRNAs

**Figure 4:**
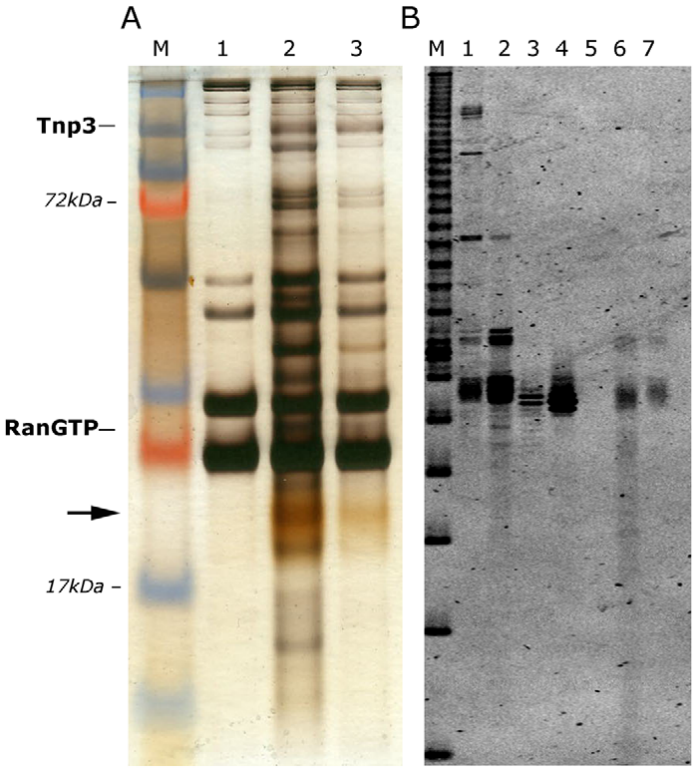
Tnp3 binds cellular tRNAs. (A) Pull down assays with high-speed cytosolic extracts (HSE) and GST-Tnp3 in the presence or absence of RanQ69L-GTP. Proteins bound to the beads were eluted and analyzed by 15% denaturing SDS-PAGE and silver staining. M, molecular weight protein markers; lane 1, beads only + HSE; lane 2, Tnp3 + HSE + RanQ69L-GTP; lane 3, Tnp3 + HSE without RanQ69L-GTP. The arrow points to the ∼20 kDa tRNA band. (B) Nucleic acids were purified from the same fractions, analyzed by 15% denaturing PAGE and visualized following staining with SYBRgold. Lane M, 10 bp ladder; Lane 1, total RNA from 293T cells; Lane 2, small RNA fraction from 293T cells; Lane 3, in vitro synthesized control tRNA; Lane 4, same tRNA x3; Lane 5, HSE; Lane 6, Tnp3 + HSE + RanQ69LGTP; Lane 7, Tnp3 + HSE without RanQ69LGTP.

The pull down assays showed greater binding of Tnp3 to viral tRNAs in the presence of RanGTP, suggesting that Tnp3 may be an export factor for these molecules. We wanted to investigate this aspect further and understand if the putative Tnp3 export activity was limited to viral tRNAs or was broader and extended to cellular tRNAs as well. To test if cellular tRNAs bound Tnp3 we performed pull-down assays with high speed cytosolic extracts (HSEs), which are devoid of ribosomes and polysomes but contain plenty of cellular tRNAs [15]. HSE was incubated with GST-Tnp3 beads and factors bound to the beads were eluted and analyzed by SDS-PAGE and silver staining. Several proteins in the HSE bound non-specifically to the GST-beads and served as a loading control but a number of other proteins specifically bound to GST-Tnp3 beads and were selectively enriched in the presence of RanQ69L-GTP (Figure 4A). Notably, a yellowish band migrating at ∼20 kDa was also specifically recovered in the Tnp3 pull downs and clearly enriched in the presence of RanQ69L-GTP (Figure 4A). This band was strikingly similar to the tRNAs band that we previously observed in HSE fractions active in HIV-1 RTC nuclear import [15], hence we purified nucleic acids in the eluted fractions and re-analyzed the samples in a long denaturing urea gel, which was stained with a fluorescent dye specific for nucleic acids. Using this procedure we could confirm that the band observed by silver staining was indeed tRNAs (Figure 4B).

**Figure 5:**
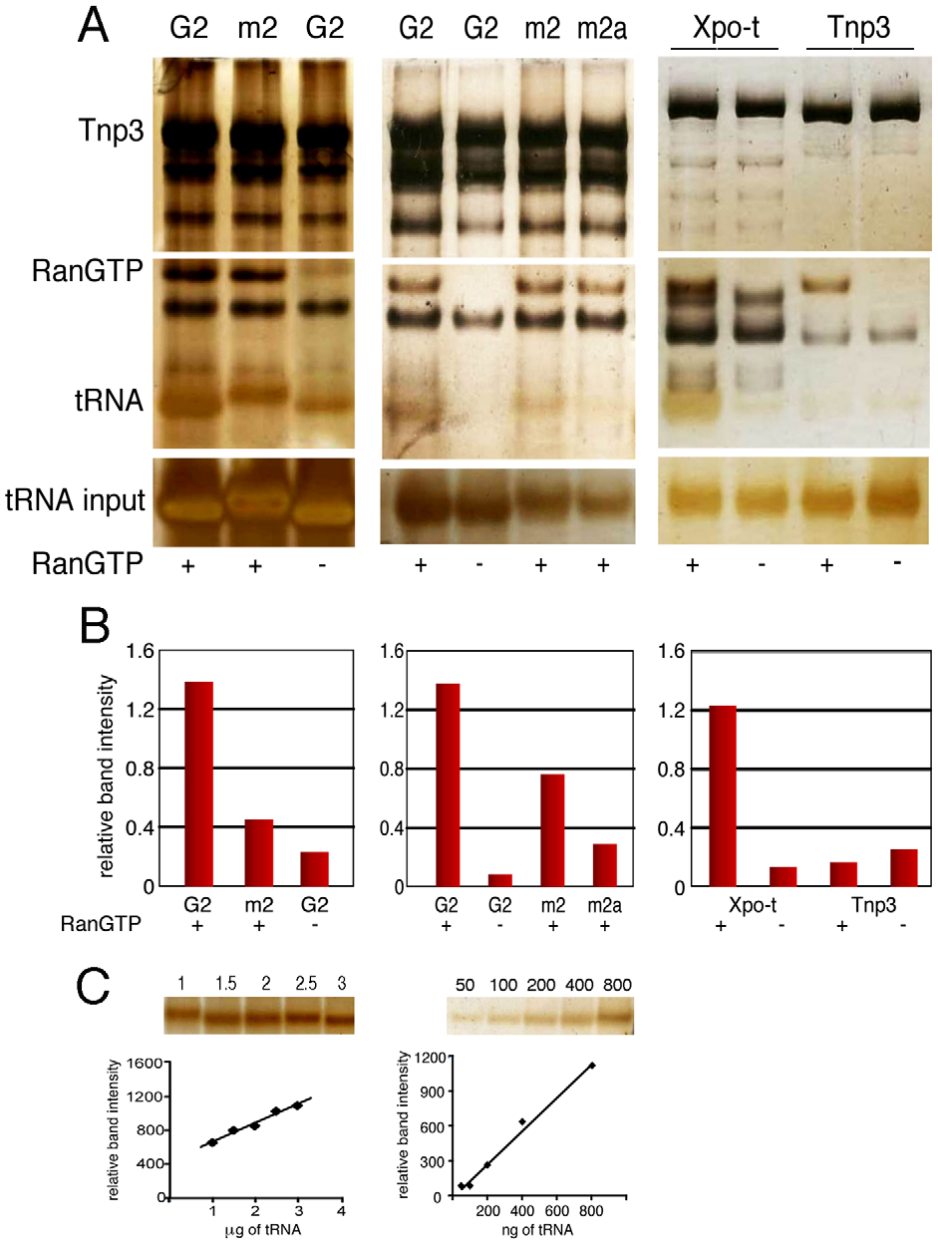
Binding of tRNAs to Tnp3 is influenced by the 3′ CCA end. (A) Left panel, pull-down assays with GST-Tnp3 and G2 tRNA specie or mutant G2 (m2) visualized by silver staining. Middle panel, pull down assays were performed with the m2 tRNA mutant or another G2 variant with a complete 3′ CCA end (m2a) (Table 1). Right panel, pull down assays with GST-Xpo-t or GST-Tnp3 and the m2a tRNA variant in the presence or absence of RanGTP. The (-) symbol at the bottom of the panels indicates that RanGDP was added in place of RanGTP. (B) ImageJ quantification of pull down assays shown in A. Values are expressed as ratio of recovered versus input tRNAs. (C) tRNA titration curves following SDS-PAGE and silver staining.

Certain tRNA species lacking a complete 3′ CCA end have been previously shown to promote HIV-1 nuclear import [15]. We therefore asked whether the same tRNAs would bind Tnp3 in the presence of RanGTP. To this end we performed pull down assays with in vitro synthesized tRNA^lys1,2^ lacking the 3′ CCA tail (hereafter called G2). To control for specificity, a mutant G2 tRNA with a 3′ TTT tail replacing the normal CCA tail (hereafter called m2) was tested in parallel and assays were performed in the presence of RanGTP or RanGDP. Tnp3 bound G2 tRNA with greater affinity in the presence of RanGTP than RanGDP, in agreement with the results obtained with cellular tRNAs. Interestingly, the m2 mutant bound Tnp3 less efficiently than G2, suggesting that the 3′ tail may modulate recognition (Figure 5A and 5B left panel).

### tRNA regions required for binding to Tnp3

**Figure 6:**
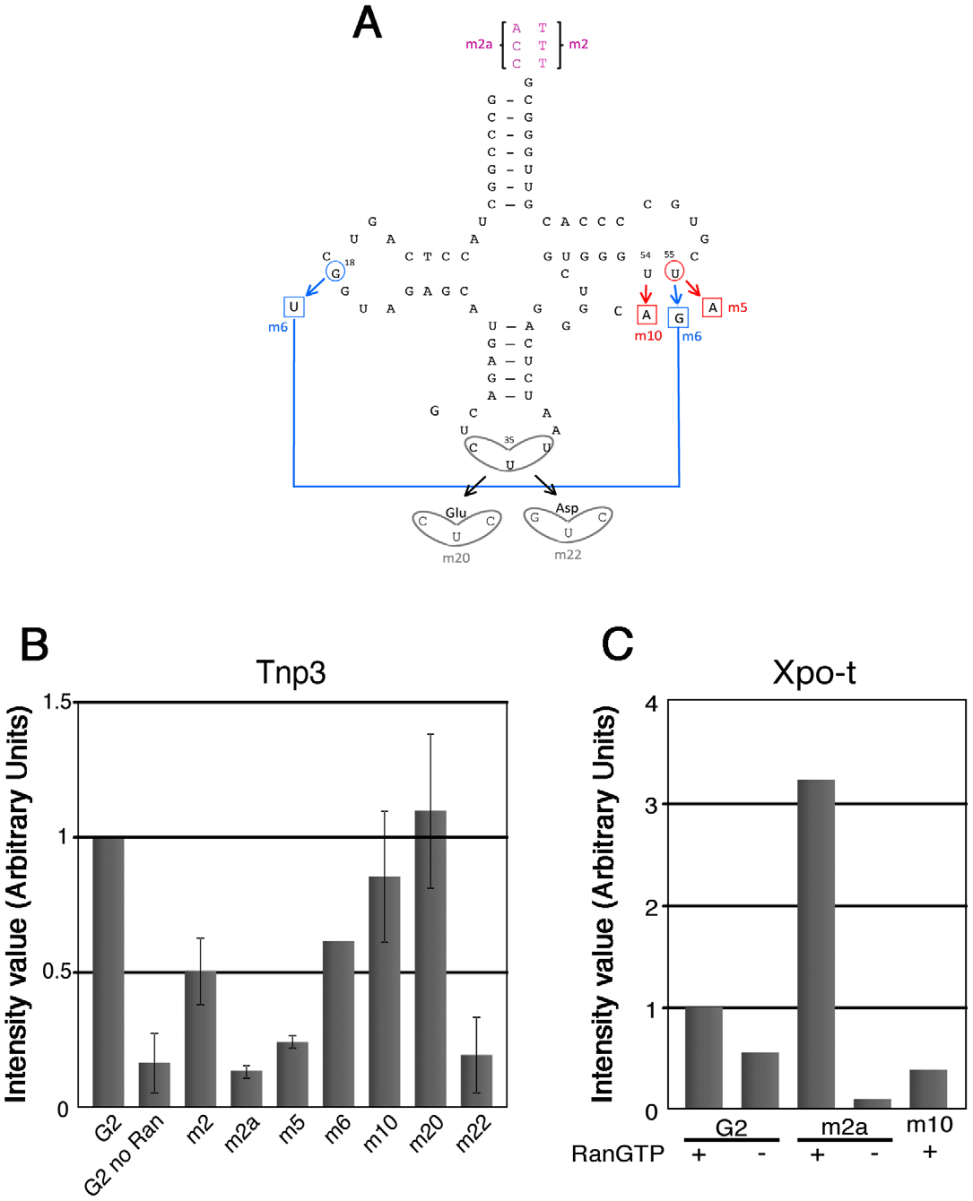
tRNA structural requirements for binding to Tnp3. (A) schematic representation of the tRNA secondary structure (cloverleaf) with the mutations generated. Nucleotide changes were introduced by site-directed mutagenesis into the G2 tRNA backbone. Mutation affecting the overall tRNA 3D structure is shown in red; a revertant of this mutation is shown in blue; anticodon swaps are indicated in grey, 3′ end additions are shown in purple. (B) Pull down assays were performed in the presence or absence of Tnp3 and RanQ69L-GTP, as indicated. Eluted tRNAs and proteins were analyzed by SDS-PAGE and silver staining and quantified by ImageJ software. Values are expressed as ratio of recovered versus input tRNAs. G2 tRNA was given an arbitrary value of 1. Values above 1 indicate the fold increase in binding and values below 1 indicate the fold decrease in binding. Mean values ± SD of three independent experiments are shown. (C) Same as panel (B) but the pull down assays were performed in the presence of Xpo-t.

To explore the role of the 3′ tail and to map other tRNA regions important for Tnp3 binding, we generated a panel of tRNA mutants and examined their ability to bind Tnp3 in pull down assays (Table 1, Figures 5 and 6). As a control, selected mutants were also tested for their ability to bind Xpo-t, which has rather stringent structural requirements for tRNA recognition, including a full 3′ CCA tail [34], [35], [36], [37]. Remarkably, a G2 variant having a complete 3′ CCA end (mutant m2a) bound Tnp3 poorly (Figure 5A and 5B, middle panels). In contrast, the m2a tRNA bound well to Xpo-t (Figure 5A and 5B, right panels). A G2 tRNA variant with the U55A point mutation in the T-loop region (m5) also showed weaker binding to Tnp3 than G2 (Figure 6). This mutation disrupts the interaction between nucleotides U55-G18, which is important for correct tRNA 3D folding [38], suggesting that a certain degree of tertiary structure integrity is necessary. This was confirmed by generating the U55G/G18U double mutant (m6) to restore the interaction between nucleotides 55 and 18, which indeed showed an improved binding to Tnp3, if compared to m5 (Figure 6). Single point mutation m10, which disturbed the T-loop conformation, did not significantly impact on binding to Tnp3 while the affinity to Xpo-t was decreased (Figure 6). These observations suggested that tRNA binding to Xpo-t is more sensitive to disruptions of the T-loop tertiary structure then Tnp3. Intriguingly, swapping the G2 anticodon sequence from lysine to aspartate (m22) inhibited tRNA binding to Tnp3 but swapping to glutamate (m20) did not (Figure 6 and Table 1). Overall these results indicated that substantial disruptions of the tRNA tertiary structure impair Tnp3 binding, and that the anticodon may be involved in recognition of Tnp3. These findings also suggest that Tnp3 selectively binds tRNAs lacking a mature 3′CCA end, in contrast to Xpo-t where the intact 3′CCA tail is required for efficient interaction – an observation which reinforces the idea that Tnp3 and Xpo-t have different requirements for tRNA recognition.

**Table 1:** tRNA mutants generated and their characteristics.

Mutant name	mutation	mutation effect
**G2**	G2 WT	Lysine 1,2 without CCA at 3′ end
**m2**	G2+TTT	G2 with TTT at 3′ end
**m2a**	+CCA	inhibited in nuclear import
**m5**	U55A	disruption of T-loop/D-loop pairing
**m6**	G18U/U55G	Functional revertant of m5
**m10**	U54A	T-loop point mutation with partial disruption of T-loop structure
**m20**	Lys->Glu	anticodon swap from Lysine to Glutamate
**m22**	Lys->Asp	anticodon swap from Lysine to Aspartate

### Domains of Tnp3 involved in tRNA binding and export

**Figure 7:**
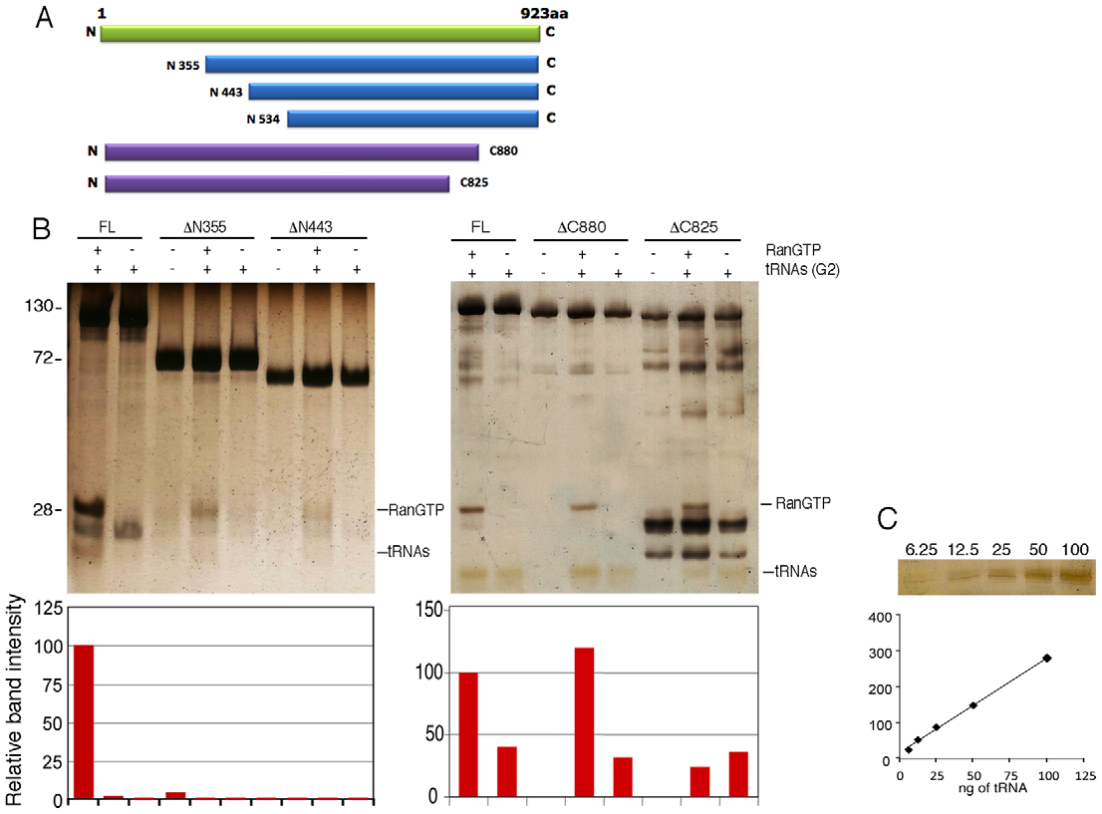
Tnp3 domains required for tRNA binding. (A) Schematic representation of the Tnp3 mutant constructs. (B) Pull down assays in the presence of the indicated Tnp3 mutants, RanQ69L-GTP and G2 tRNA. Eluted proteins and tRNAs were analyzed by 15% SDS-PAGE and silver staining. Smaller bands <100 kDa are degradation impurities of Tnp3. Molecular weights (kDa) are shown on the left. ImageJ quantification of tRNA band intensity is shown below each panel. (C) tRNA titration curves following SDS-PAGE and silver staining.

To map the Tnp3 domains important for tRNA binding, several N- and C- terminal Tnp3 deletion mutants were generated and tested in pull down assays using equimolar amounts of tRNAs and Tnp3 for cross-comparison (Figure 7). A deletion in the first 355 N-terminal residues of Tnp3 significantly inhibited binding to RanGTP, in agreement with a previous study [39], and to tRNAs. An even greater loss of tRNA binding was observed when the first 443 N-terminal residues of Tnp3 were deleted (Figure 7B). Unfortunately, further N-term Tnp3 deletion mutants were poorly soluble and could not be tested. Nonetheless, these results clearly showed that a critical RanGTP binding domain was located within the first 355 N-terminal residues of Tnp3, with residual binding up to the first 443 N-terminal residues, and that binding of RanGTP was important for tRNA recognition.

Two C-terminal Tnp3 deletion mutants were generated, DC880 and DC825. Pull down assays with these two mutants showed that binding of tRNA was mainly dependent on residues between 825 and 880 of Tnp3 (Figure 7B – right panel). Interestingly, the DC880 and DC825 Tnp3 mutants lost some affinity for RanGTP, suggesting that Tnp3 may form a ternary complex similar to the one recently described for Xpo-t, in which RanGTP makes contact with the C-term of Xpo-t [36]. These results are also consistent with the pull down results obtained using viral tRNAs (Figure 3C and Figure 3D)

**Figure 8:**
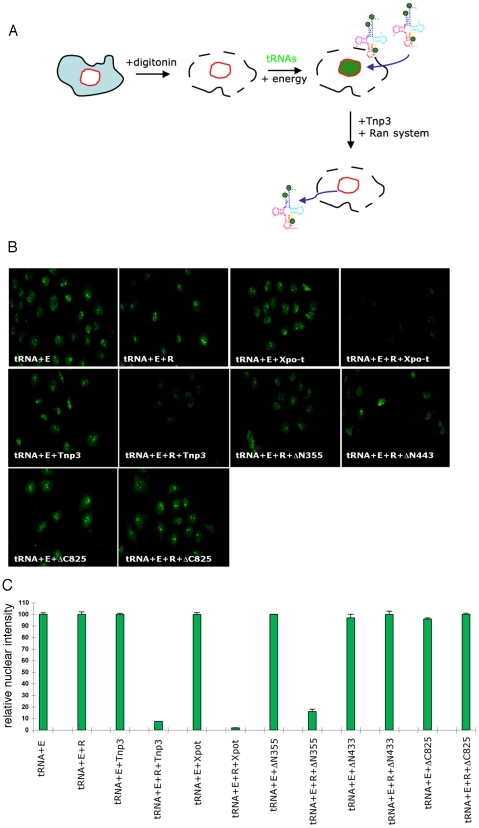
Tnp3 is an export receptor for certain tRNA species. (A) Schematic representation of the nuclear export assay. Following cell permeabilization, a nuclear import step is performed with fluorescently labeled tRNAs and the energy regenerating system. Once tRNAs accumulated into the nucleus, cells are washed and a second incubation is performed in the presence of Tnp3, the Ran system and the energy system. Samples are washed and analysed by confocal microscopy. Loss of fluorescence relative to control without Tnp3 indicated tRNA export. (B) Nuclear export of fluorescently–labelled G2 tRNAs (∼100 ng each assay) in permeabilized HeLa cells in the presence of an energy-regenerating system (E), the Ran system (R) and 1 µM of the indicated recombinant proteins. (C) Quantification of tRNA nuclear export. Images acquired by confocal microscopy were analyzed by MetaMorph software version 4.5r4 and the total nuclear fluorescence divided by the number of cells per field. Bars represent the mean fluorescence per nucleus ± average deviation of two independent experiments. At least 100 cells were counted per experiment.

Export of tRNAs from the nucleus is a quality controlled process, whereby Xpo-t selectively binds fully mature tRNAs suitable for protein translation [34], [35]. It was therefore surprising to find that Tnp3 might be an export factor for defective tRNAs lacking a complete 3′ CCA end, which hinted at the possibility that a parallel tRNA export pathway might exist in mammalian cells. To examine if Tnp3 could indeed export these tRNA species, an export assay was devised in permeabilized HeLa cells by adapting the classical nuclear import assay [40] (Figure 8A). To this end, HeLa cells were permeabilized by digitonin and a standard nuclear import assay was carried out first in the presence of purified and fluorescently labeled tRNAs (G2) and an energy regenerating system. After 10 minutes incubation at 37°C to allow tRNA nuclear accumulation [15], cells were washed and incubated for a further 10 minutes at 37°C in the presence or absence of recombinant Tnp3, an energy regenerating system and the Ran system. Following this second incubation, cells were washed, fixed and analyzed by confocal microscopy. A tRNA export receptor should reduce the fluorescent signal from pre-loaded nuclei and relocate tRNAs to the cytoplasmic side where they are washed out. This modified assay was initially tested with Xpo-t and provided an excellent readout for tRNA export (Figure 8B).

**Figure 9:**
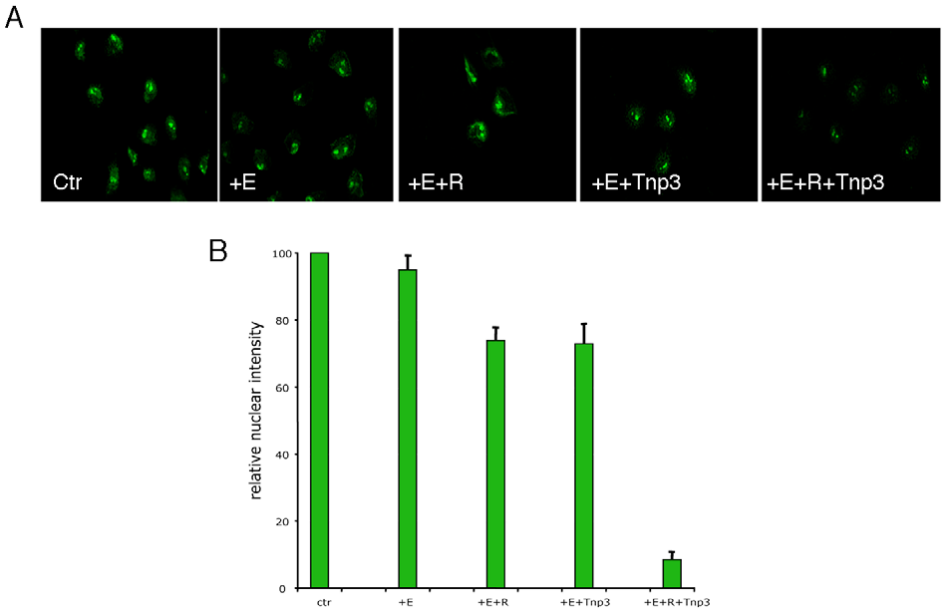
Tnp3 is an export factor for tRNAs incorporated into HIV-1 particles. (A) Viral tRNAs were isolated from purified HIV-1 particles (as shown in Figure 3A), fluorescently labeled and used (∼100 ng each assay) in the nuclear export assay as described in Figure 8 in the presence of an energy-regenerating system (E), the Ran system (R) and 1 µM Tnp3. (B) Images acquired by confocal microscopy were analyzed by MetaMorph software version 4.5r4 and the total nuclear fluorescence divided by the number of cells per field. Bars represent the mean fluorescence per nucleus ± average deviation of two independent experiments. At least 100 cells were counted per experiment.

In the export assay, addition of Tnp3 clearly reduced the nuclear fluorescent intensity compared to controls and the effect was specific because it was observed only in the presence of the Ran system (Figure 8B). The reduction in the fluorescent signal could not be explained by contamination of the protein preparation with RNAse because it was not observed upon addition of the individual components (Figure 8B, 8C). Moreover, the export activity of the DN355, DN443 and DC825 Tnp3 deletion mutants correlated with their affinity for RanGTP and tRNAs as detected in pull down assays (Figure 8B, 8C). We found that the fluorescent signal was reduced in both nuclei and cytoplasm, presumably because tRNAs in complex with Tnp3 were more soluble and could be more easily removed from the cytoplasmic remnants of permeabilized cells by washing. These results confirmed that Tnp3 is a RanGTP-dependent export factor for some tRNAs species lacking a complete 3′ CCA end. To test if endogenous viral tRNAs were also exported by Tnp3, we extracted tRNAs from purified viral particles and used them in the export assay. Similarly to the in vitro synthesized G2 tRNA, endogenous viral tRNAs were also exported by Tnp3 in the presence of the Ran system (Figure 9).

### HIV-1 CA is an important determinant for Tnp3 activity

**Figure 10:**
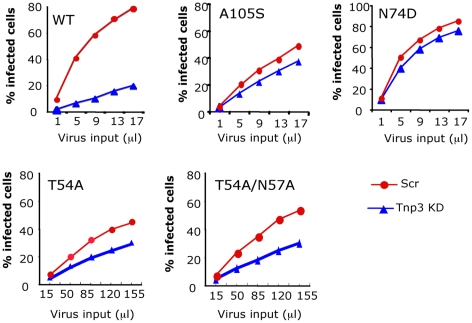
CA is an important determinant for HIV-1 dependence on Tnp3. Infection assays were performed in stable HeLa Tnp3 KD cells or control cells expressing a scrambled shRNA using HIV-1_GFP_ vectors bearing the indicated mutation in CA. Viral stocks were normalized for RT activity and different volumes used for titrations. Infected cells were analysed by flow cytometry 48 hours post-infection. Note that the T54A and T54A/N57A have ∼20 fold lower infectivity than wild type virus. Data are representative of at least 3 independent experiments.

In pull down assays with purified HIV-1 vector we found that Tnp3 bound to CA, suggesting that it is an important target for Tnp3 function. To further investigate the relevance of CA in influencing susceptibility to Tnp3, we generated a panel of HIV-1 vectors with mutations in CA, which were previously shown to be defective for infection at a post-nuclear entry step [11] and examined their dependence on Tnp3 in stable HeLa Tnp3 KD cells (Figure 10). The N74D point mutant, which is independent of Tnp3 for infection [14] was tested in parallel. The T54A and the T54A/N57A capsid mutant HIV-1 vectors showed substantially reduced infectivity compared to wild type virus, however they were also less dependent on Tnp3 for infection (Figure 10). We also tested the A105S mutation, which conferred resistance to the antiretroviral compound Coumermycin-A1 [41]. Interestingly, Coumermycin-A1 was shown to inhibit HIV-1 integration [41], similarly to the phenotype observed in Tnp3 KD cells, suggesting that the compound and Tnp3 may act on same pathway. The A105S mutant vector maintained near-wild type infectivity and was also clearly less dependent on Tnp3 for infection. Of all mutant vectors the N74D was the least dependent on Tnp3 for infection and maintained normal infectivity (Figure 10). Similar results were obtained using an HIV-1 vector bearing the gp120 envelope (Figure S4). These results confirmed that CA is an important target of Tnp3 [14], [19].

### Tnp3 is an export factor for CA

**Figure 11:**
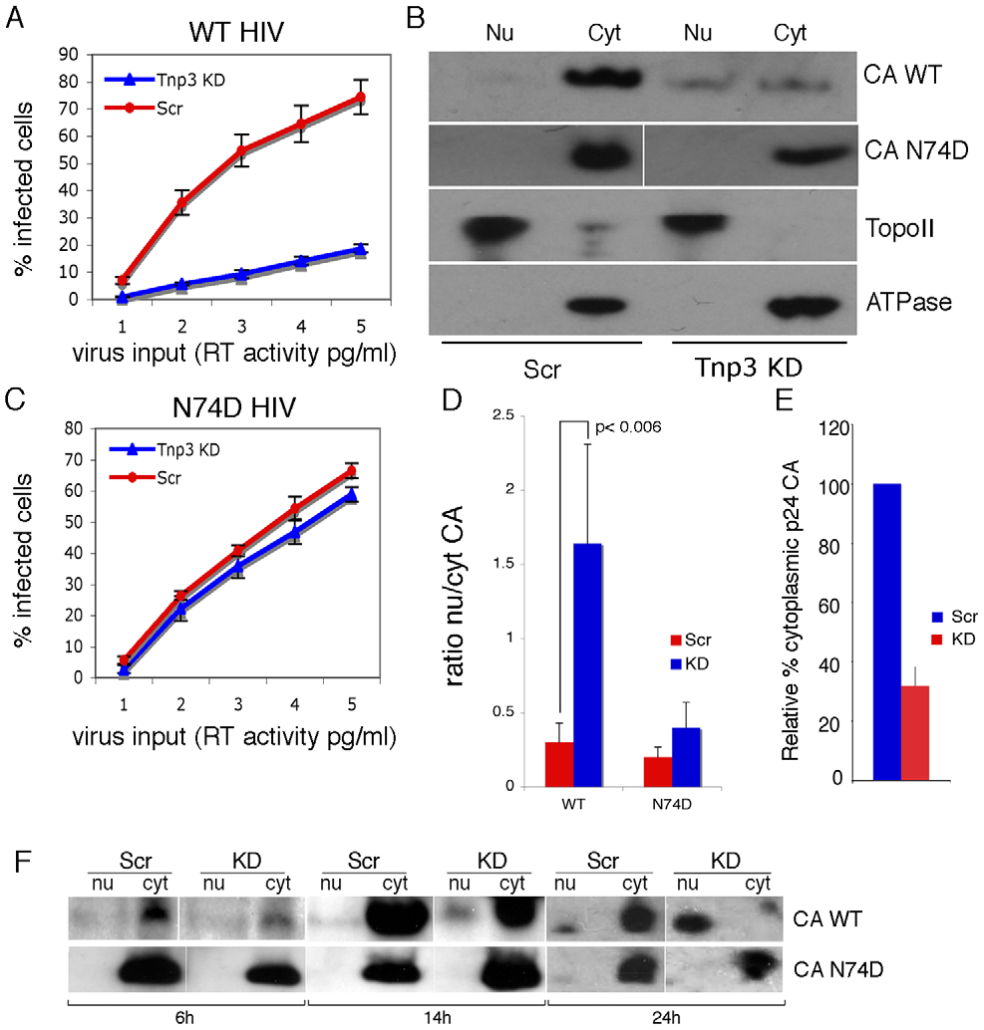
Tnp3 affects the nucleo-cytoplasmic distribution of CA. (**A**) Control (Scr) and Tnp3 KD HeLa cells were infected with WT HIV-1_GFP_ vector and analyzed by flow cytometry 24 hours later; mean values ± SD of three independent experiments are shown. (**B**) Infected cells were fractionated into nuclear and cytoplasmic fractions 16 hours post-infection and each fraction was analyzed by Western blot. N+/K+ ATPase (ATPase) and DNA topoisomerase II (TopoII) were used to control the fractionation procedure. Data are representative of three independent experiments. (**C**) Control (Scr) and Tnp3 KD HeLa cells were infected with the N74D mutant vector and analyzed by flow cytometry 24 hours later; mean values ± SD of three independent experiments are shown. (**D**) Relative amount of CA detected by Western blot in nuclear and cytoplasmic fractions was quantified by ImageJ; mean values ± SD of three independent experiments are shown. (E) Relative amount of CA detected by Western blot in the cytoplasmic fractions of Scr and Tnp3 KD cells was quantified by ImageJ; mean values ± SD of three independent experiments are shown. (F) Time course of CA nuclear accumulation. Cells were transduced with the HIV-1_GFP_ vector at an MOI of 0.5 and fractionated at the indicated time points. Fractions were analysed by Western blotting with anti-CA antibodies. Virus input was normalized for infectivity in HeLa cells, hence the higher amount of N74D CA detected at the 6h time point. Similar results were obtained in another independent experiment.

Next we examined what the functional significance of the interaction between CA and Tnp3 might be. The pull down assays showed that Tnp3 binding to CA was stronger in the presence of RanGTP. We therefore asked if Tnp3 was required for the export of capsid from the nucleus of infected cells. We could not detect accumulation of recombinant HIV-1 CA into the nucleus of permeabilized cells, precluding the use of the nuclear export assay in this case. Therefore, control and stable Tnp3 KD cells were transduced at an MOI of 0.5 with the HIV-1 vector (Figure 11A), fractionated into nuclear and cytoplasmic fractions 16 hours post-infection and the distribution of CA in each fraction was examined by Western blot (Figure 11B). A relatively low MOI was used because control and Tnp3 KD cells maintained a clear phenotype with respect to HIV-1_GFP_ infection in these conditions. Following fractionation, CA was detected in the nuclear fraction of Tnp3 KD cells and, to a lesser extent, control cells, whereas cytoplasmic CA was significantly less abundant in Tnp3 KD than control cells (Figure 11E). The biological significance of this result was confirmed using an HIV-1 vector bearing the N74D mutation in CA, which is the least dependent on Tnp3 for infection [14] (Figure 11B, 11C). The N74D mutant virus did not appear to accumulate CA in the nuclei in either control or Tnp3 KD cells (Figure 11B). The ratio of nuclear to cytoplasmic CA was significantly higher in KD than control cells infected with wild type virus but remained essentially unchanged in cells infected with the N74D mutant (Figure 11D). A different intracellular distribution of wild type and N74D CA was also observed by immunofluorescence (Figure S5). In this case, however, we had to increase the MOI by 10 fold to ≥5 to be able to see a specific intracellular p24 signal, which resulted in an almost complete loss of phenotype in Tnp3 KD cells relative to control cells upon infection with the HIV vector.

To investigate if the temporal dynamics of nuclear accumulation of CA coincided with that of integration, we performed a time-course experiment in cells infected with the HIV-1_GFP_ vector. Trace amounts of CA could be detected 6 h post-infection, then CA progressively accumulated into the nucleus of Tnp3 KD cells at 14 h and 24 h post-infection (Figure 11F). Significantly less CA was detected in the nucleus of control cells 14 h and 24 h post-infection compared to KD cells (Figure 11F). In contrast, the N74D CA mutant was not found accumulating into the nuclei of either control or Tnp3 KD cells (Figure 11F). The timing of CA nuclear accumulation was consistent with the dynamics of viral integration in single-cycle infection assays [27], [42], supporting the notion that Tnp3 promotes a maturation step, possibly by displacing any CA and tRNAs still bound to the PIC.

## Discussion

The steps of the HIV-1 life cycle between nuclear import and integration are incompletely understood, in part due to technical challenges and in part because much of the relevant molecular cell biology of nuclear transport, chromatin remodeling and nuclear dynamics have been discovered more recently. Greater knowledge of these early steps of HIV-1 infection will impact on the development of drugs with novel mechanisms of action and will shed light on more fundamental cellular processes. Here we have found evidence for an unanticipated step in the HIV-1 life cycle occurring in the nucleus of infected cells that depends on the cellular factor Tnp3. Connected to this step, we have also found evidence for a novel cellular pathway dedicated to tRNA nucleocytoplasmic shuttling.

In our experimental conditions Tnp3 was mainly required for efficient HIV-1 integration. This may appear to contradict previous studies [18], [20], [21], however Brass et al. reported a significant integration defect but did not show any data on nuclear import in Tnp3 KD HeLa TZM-bl cells [18] and König et al. reported a significant drop in the number of integrated proviral copies in Tnp3 KD 293 cells but no changes in 2LTRs [21]. Christ et al. detected a modest (2–3 fold) block to 2LTR circular DNA formation in Tnp3 KD HeLaP4 cells but observed a substantially greater defect in viral integration [20]. Although presently we do not exclude some effect on HIV-1 nuclear entry, we can however conclude that integration is indeed an independent and important step blocked in cells depleted of Tnp3.

### Tnp3, nucleocytoplasmic shuttling of tRNAs and HIV-1 infection

Tnp3 was previously shown to bind to HIV-1 IN [20], which provided an attractive explanation for the infectivity defect observed in Tnp3 KD cells. However the physiological relevance of the Tnp3/IN interaction remains uncertain, and the ability of Tnp3 to support HIV-1 infection mainly maps to CA [14], [19]. Therefore we have taken an unbiased approach and performed pull down assays to examine which components bind to Tnp3 in the context of a mature viral particle. We detected binding of Tnp3 to CA and viral tRNAs and, to our surprise, the affinity of the interaction was greater in the presence of RanGTP (Figure 3). Tnp3 is a nuclear import receptor for some serine-arginine rich (SR) proteins, such as the splicing factor ASF and the human papilloma virus-5 (HPV-5) E2 protein, which are released upon RanGTP binding [39], [43]. The observation that Tnp3 bound viral tRNAs better in the presence of RanGTP suggested that it has both nuclear import and nuclear export activity, depending on the cargo. Other examples of this dual activity include importin 13 [44], exportin 4 [45], [46] and Mtr10p [47], [48], the hortologue of Tnp3 in *S. cervisiae*. Tnp3 bound and exported some cellular tRNAs as well as in vitro synthesized tRNAs lacking the 3′ CCA end. In this respect Tnp3 differs from Xpo-t, which binds preferentially mature tRNAs with a complete 3′ CCA end [34], [35], [36]. These differences are most likely related to function: Xpo-t is the main nuclear export receptor for mature tRNAs that participate in protein translation, whereas Tnp3 appears more likely to be involved in nuclear export of “defective” tRNAs. Tnp3 may be more similar to exportin 5 (exp5), which has more relaxed structural requirements, recognizing and exporting different double stranded RNAs, including tRNAs and miRNAs [49], [50], [51], [52], [53].

Recent evidence showed that regulation of the tRNA retrograde pathway is more complex than anticipated in *S. cervisiae* [16], [54], [55]. Whereas active nuclear import of tRNAs is constitutive, export of tRNAs is regulated by availability of nutrients and inorganic phosphates [56], [57]. At least two parallel tRNAs export pathways have been detected: one for the initial and subsequent export of mature tRNAs, mediated by Los1p (the yeast hortologue of Xpo-t), and a secondary export pathway, mainly mediated by Msn5, the yeast hortologue of exportin 5 [58], [59]. A similar scenario might be present in human cells, whereby several parallel tRNA export pathways serve different purposes. Although Mtr10p is implicated in tRNA nuclear import in yeast [54], [59], Tnp3 appears to be mainly implicated in export of “defective” tRNAs in human cells. Future work will elucidate the basis for this difference and if additional co-factors participate in the tRNA shuttling process in human cells. It is noteworthy that, in human cells, we have observed energy-dependent nuclear accumulation primarily of defective tRNAs lacking a complete 3′ CCA end [15]. However, whereas nuclear import of defective tRNAs may serve as a quality control process by withdrawing such tRNAs from the protein synthesis machinery, it is less clear at present why defective tRNAs should also be exported from the nucleus of human cells. One possibility is that defective tRNAs serve specific purposes, distinct from protein translation. Supporting this hypothesis, there is growing evidence showing that truncated or fragmented tRNAs are specifically generated in mammalian cells and contribute to the regulation of protein synthesis, cell division and cell growth [60], [61], [62], [63], [64]. Hence shuttling truncated tRNAs in and out of the nucleus may have a fundamental biological function, quite separate from HIV-1 infection. Another possibility is that shuttling to a different cellular compartment signals that a defective tRNA must be repaired.

Tnp3 binding to tRNAs was influenced by structural features such as the lack of the complete 3′ CCA tail, similar to tRNA nuclear import in human cells [15], suggesting that the two processes may be linked. Similar structural requirements for tRNA retrograde transport on the one hand and Tnp3-mediated tRNA nuclear export on the other hand as well as the involvement of CA proteins in both processes [14], [15] point to a common pathway of which import and export may be two sides of the same coin.

It may seem counter-intuitive for HIV-1 to depend on defective tRNAs for replication. However in metabolically active cells such as chick embryo fibroblasts, the rate of tRNA synthesis has been estimated at ∼2.4×10^5^ molecules/min/diploid genome, with a steady state value of ∼10^9^ tRNA molecules/diploid genome [65]. The tRNA half-life in dividing cells ranges between 50 h to 60 h [65], [66]. Importantly, the 3′ CCA end is the most exposed portion of the tRNA molecule and the most sensitive to a variety of nucleases because it is single stranded. Therefore, the rate of tRNA molecules being hydrolyzed at the 3′ CCA end is likely to be very high yet steady state levels of tRNAs with defective ends are by far lower than normal tRNAs. The simplest explanation for this is that tRNA repair/degradation mechanisms, which presumably include tRNA shuttling, are *extremely* efficient. Therefore HIV-1 may in fact have evolved to use one of the most efficient cellular trafficking pathways available.

### Tnp3 modulates nuclear “uncoating”

In addition to tRNAs, Tnp3 also bound HIV-1 CA in pull down assays. The strength of this interaction was greater in the presence of RanGTP, suggesting that CA may be an export cargo for Tnp3. The permeabilized cell assay could not be used to investigate this possibility because we could not detect nuclear import of recombinant CA. We therefore examined the nucleo-cytoplasmic distribution of CA after acute infection of Tnp3 KD and control cells. Remarkably, Tnp3 KD cells contained lower amounts of cytoplasmic CA and relatively greater amounts of nuclear CA compared to control cells. We also detected reduced levels of total (nuclear + cytoplasmic) CA in Tnp3 KD cells compared to controls. The reason for this is unclear but CA retained into the nucleus due to lack of Tnp3 may be targeted for rapid degradation in dividing cells, similarly to other nuclear factors [67]. A time course experiment showed that the dynamics of CA nuclear accumulation appear to overlap with that of HIV-1 integration in single cycle assays [27], [42]. The simplest interpretation of this result is that Tnp3 exports CA from the nucleus of infected cells. The biological significance of this result is supported by experiments with the N74D CA mutant HIV-1 vector. Among the CA mutants tested, the N74D was the least dependent on Tnp3 for infection, suggesting that either the N74D CA was shed before nuclear entry or that it could exploit different factors for its export, becoming more promiscuous. In pull down assays, we have detected binding of Tnp3 to the N74D CA mutant (Figure S5). We have also detected CA in the nuclei of control cells 16 h post-infection by immunofluorescence whereas mutant N74D CA localized mostly outside the nuclei or at the nuclear envelope (Figure S5). The time course experiment shown in Figure 11 also suggested that N74D CA does not enter into the nucleus, at least for up to 24 h post-infection. Therefore it appears that the N74D CA is shed before nuclear entry of the RTC/PIC, making Tnp3 unnecessary. It will be interesting to see if such a premature loss of CA impacts on other downstream events.

Accumulating evidence indicates that HIV-1 CA impacts on post-nuclear entry events [9], [17], [68]. Furthermore, a functional link between HIV-1 CA and integration has been recently described using a chemical genetic approach, whereby the small molecule Coumermycin-A1 impaired integration by targeting HIV-1 CA [41]. Interestingly, the A105**S** CA mutation made the virus insensitive to this block [41]. It is remarkable that the same mutation also makes HIV-1 independent of Tnp3 for infection, suggesting that Coumermycin-A1 and lack of Tnp3 perturb the same pathway. Moreover, the A105**T** mutation in CA was shown before to influence HIV-1 post-entry events in a cyclophilin A (CypA) dependent way [68]. However the mechanism by which CA impacts on post-nuclear entry events and integration is poorly understood. One hypothesis is that insufficient uncoating of the viral core in the cytoplasm may affect downstream events by making the pre-integration complex too bulky or unsuitable to bind specific host factors. Alternatively, following uncoating in the cytoplasm, some CA may remain bound to the pre-integration complex and help HIV-1 negotiate through the NPC [14], [69]. In this case, some CA is likely to remain bound to the PIC after nuclear translocation. Indeed the presence of CA associated with the PIC inside the nucleus can be inferred from previous studies in which the restriction factors Fv-1 and members of the TRIM protein family were fused to CypA. The resulting fusion proteins maintained their specific ability to bind CA yet restricted HIV-1 at a post-nuclear entry step [70], [71].

**Figure 12:**
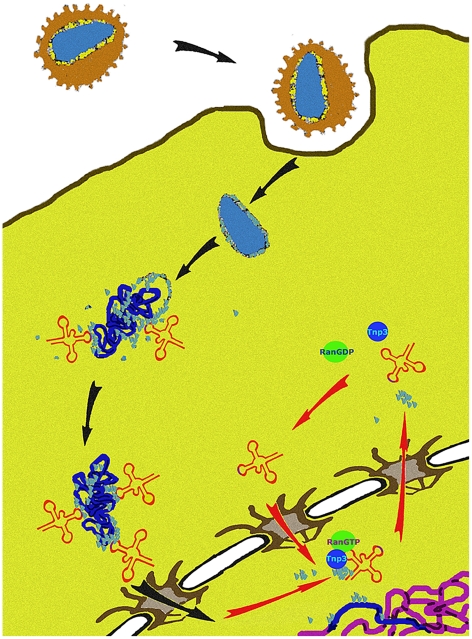
A model of the role of Tnp3 in HIV-1 infection. After virus entry into the cell, the viral core is disassembled but some capsid proteins (light blue dots) remain associated with the RTC. A complex is formed between capsid proteins, tRNAs and presumably one or more host cell factors to engage with the tRNA retrograde transport pathway, leading to nuclear import of the RTC/PIC. CA associated to the RTC/PIC may also play a role in nuclear import by binding to components of the NPC, as well as other factors that may associate with IN. Once inside the nucleus, the PIC must complete the uncoating process by removing any remaining capsid proteins and tRNAs, which are detrimental to integration. Tnp3, in complex with RanGTP, carries out this step.

We propose a unifying hypothesis. Our results show that Tnp3 promotes the export of certain viral components from the nucleus of infected cells and that efficient HIV-1 integration depends on this activity. Therefore, several uncoating steps may be necessary for HIV-1 infection. The main uncoating step likely occurs in the cytoplasm, presumably starting shortly after initiation of reverse transcription [6], [10], and the last uncoating step occurs inside the nucleus. To permit efficient nuclear entry, some CA and tRNAs must remain associated with the viral complex [14], [15] but once their function is exhausted, these same elements must be displaced to facilitate integration (Figure 12). We propose that Tnp3 is the displacing factor. The requirement for RanGTP ensures that Tnp3 binds to the viral elements with greater affinity after the PIC has entered into the nucleus, where RanGTP concentration is highest [24]. PICs that have not “matured” may fail to interact with host factors present in the nucleus, impairing integration both quantitatively and qualitatively. Our model is supported by recent evidence indicating that depletion of Tnp3 results in an aberrant pattern of HIV-1 integration [72].

## Materials and Methods

### Ethics statement

Blood was obtained from healthy volunteers after written informed consent according to the approved protocol of the UCL Ethics Committee ref. 0335/001 or from buffy coats obtained from the NHS National Blood Service according to Governmental ethics regulations.

### Cell culture, virus production and infection

HUES-2 hES cells [73] were differentiated to monocytes via embryoid body formation followed by directed differentiation in Advanced DMEM, supplemented with 10% FCS, 100 ng/mL M-CSF (R&D), 25 ng/mL IL-3 (R&D), 2 mM l-glutamine (Invitrogen GIBCO), 100 U/mL penicillin and 100 µg/mL streptomycin (Invitrogen GIBCO), and 0.055 mM β-mercaptoethanol (Invitrogen GIBCO). Monocytes emerging into the supernatant after 3–6 weeks were harvested and further differentiated to macrophages for 1 week at a density of 1.5×10^5^ cells/cm^2^, in culture medium consisting of RPMI (Invitrogen GIBCO) supplemented with 10% FCS, 100 ng/mL M-CSF (R&D), 2 mM l-glutamine (Invitrogen GIBCO), 100 U/mL penicillin and 100 µg/mL streptomycin (Invitrogen GIBCO) [25]. Macrophages were isolated from buffy coats from healthy donors by standard Ficoll-Hypaque density centrifugation. Cells were incubated at a density of 5×10^4^/well in a volume of 100 µL in 96 well plates for 72 h in the presence of 20 ng/ml of GM-CSF before media change and infection at day 5. HeLa and 293T cells were grown in Dulbecco's modified Eagle's medium (DMEM) (Gibco Labs, Paisley, UK) supplemented with 10% foetal calf serum (FCS) (Helena Bioscience, Newcastle, UK) and 2 mM glutamine at 37°C in 5% CO_2_. Jurkat cells were grown in RPMI medium supplemented with 10% FCS at 37°C in 10% CO_2_. Supernatants containing HIV-1 pseudotyped with amphotropic envelope were collected from stable producer cells [31], centrifuged at 3000 *g* for 10 mins, filtered through a 0.45 µm filter and purified as described [15]. pHIV_LAI_Δenv and HIV-1_GFP_ vectors were made and purified as described previously [2], [26]. For HIV-1_GFP_ plasmids pCSGW, pCMVΔR8.2, (expressing *gag-pol*) and pMD.G expressing VSV-G were used [12], [74], [75]; pHIV_LAI_Δenv was pseudotyped using plasmid pMD.G. Reverse transcriptase (RT) activity was measured by the Lenti-RT™ Activity Assay (Cavidi Tech, Uppsala, Sweden) following the manufacturer's instructions. For infections, HeLa cells were plated onto 24 well plates at 3×10^4^/well, infected 24 hours later with serial dilutions of viral stocks and analyzed by FACS from 24 hours to 2 weeks after infection. Jurkat cells were plated at 5×10^5^/ml in 48-well plates, infected with serial dilutions of HIV_GFP_ and analysed by FACS 48 hours later. For DNA analysis, Jurkat cells were plated at 5×10^5^/ml in 6-well plates, infected at an MOI of 0.1 and DNA extracted 24 h, 48 h and 10 days later.

### Depletion and rescue of Tnp3

Tnp3 was knocked down using two siRNAs; Tnp3-1 sense, GCAGUGAUAUUUAGGCAUAUU, antisense, UAUGCCUAAAUAUCACUGCUU; Tnp3-2 sense, GUACCAAAACUAACGAUGAAUU; antisense, UUCAUCGUUAGUUUUGUACUU [18]. HeLa cells were plated in 6-well plates at 1.5×10^5^/well and transfected with siRNAs and oligofectamine (Invitrogen) the following day according to the manufacturer's instructions. To generate stable Tnp3 KD cells, Tnp3 oligonucleotides 5′-CTAGTCGGCGCACAGAAATTATACTTCCTGTCATATAATTTCTGTGCGCCGTTTTT-3′ and 5′-GTACAAAAATCGGCGCACAGAAATTATATGACAG GAAGTATAATTTCTGTGCGCCGA-3′ or scrambled oligonucleotides 5′-CTAGTCGGCGCAGTCTAATTATACTTCCTGTCATATAATTAGACTGCGCCGATTTTT-3′ and 5′-GTACAAAAATCGGCGCAGTCTAATTATATGACAGGAAGTATAATTAGACTGCGCCGA-3′ [20] were annealed and cloned into pSIREN vector (Clontech). The resulting vector was used for virus production and infection of target HeLa, Jurkat and 293T cells. Differentiated macrophages were transduced with a lentiviral vector bearing the same shRNA expression construct. A silent mutation was introduced into the human Tnp3 cDNA to make it resistant to shRNA targeting by QuickChange II XL (Stratagene) following the manufacturer's instruction with primers C498A forward CGAATTGGAGCTAATCGGCGAACAGAAATTATAGAAGATTTG and C498A reverse CAAATCTTCTATAATTTCTGTTCGCCGATTAGCTCCAATTCG. Four clones were confirmed by sequencing and cloned into pCDNA3 to generate pTnp3-R for expression into mammalian cells. pTnp3-R was used as a template to generate ΔC825Tnp3-R by PCR using primers ΔC forward ATCATCGAATTCATGGAAGGAGCAAAGCCG and ΔC reverse ATCATCCTCGAGTCACACCTGTCCAATCAG. Clones were confirmed by sequencing. For the rescue assay, 293T cells were plated into 48-well plates at 2×10^5^/well and infected the next day with serial dilutions of pSIREN shRNA-Tnp3 or pSIREN shRNA-Scramble MLV vector. Forty-eight hours post-infection, cells were transferred into 24-well plates and selected with 1 µg/ml puromycin. Puromycin-resistant cells were plated into 6 well plates at 10^6^/well and transfected 24 h later with 1 µg and 2 µg Tnp3-R or ΔC825Tnp3-R plasmids complexed with Fugene-6 (Roche). Cells were incubated for 48 h, infected with the HIV-1 vector and analyzed by FACS 24 h post-infection.

### Generation, expression and purification of recombinant proteins

The GST-Xpo-t expression plasmid was generated by PCR from pQE30-Xpo-t [37] as a template using forward primer 5′-ATCGGATCCATGGATGAACAGGCTCTATTAG-3′ and reverse primer 5′-. ATCGAATTCTCAGGGCTTTGCTCTCTG-3′, and cloned into BamHI and EcoRI sites of pGEX-2T. GST-Xpo-t and GST-Tnp3 (pGST-TRN-SR2 [39]) plasmids were expressed in *E. Coli* strain BL21(DE3) by overnight incubation at 16°C on induction with 0.5 mM isopropyl ß-D-thiogalactoside. The GST-fusion proteins were purified by using glutathione-Sepharose beads and then dialyzed against binding buffer using a 50 kDa c/o membrane. Alternatively, GST was cleaved by incubating for 16 h at 4°C with 15 U/µg Thrombin (Amersham Biosciences) and eluted untagged proteins were dialyzed (50 kDa c/o) in import buffer. Tnp3 deletion mutants were generated by PCR with Pfu DNA polymerase using plasmid pGST-TRN-SR2 [39] as template and the following primers:

DN443 Tnp3 forward ATCGGATCCATGGCTGCTATAGCAAAGAG

DN534 Tnp3 forward ATCGGATCCATGGCTCAGCACTTTAATG

and reverse: CCGGAATTCTCATCGAAACAACCTGGTG

DC880 Tnp3 reverse CCGGAATTCTCATGGCAAACCTTTTAAGG

DC825 Tnp3 reverse CCGGAATTCTCACACCTGTCCAATCAG

and Tnp3 forward ATCGGATCCATGGAAGGAGCAAAGC

PCR products were cloned into pGEX-2T using BamHI and EcoRI, confirmed by sequencing and expressed in *E. Coli* strain BL21(DE3) by overnight incubation at 16°C on induction with 0.5 mM isopropyl ß-D-thiogalactoside. The GST-fusion proteins were purified by using glutathione-Sepharose beads and by gel filtration using a Superdex 200 column. The components of the Ran system and RanQ69L were expressed and charged with GTP or GDP as previously described [30], [76], [77].

### Preparation of 60S HSE and viral samples

The pellet from ∼10^9^ HeLa cells was washed once in phosphate-buffered saline (PBS) and resuspended in 5 vols of hypotonic buffer (10 mM Hepes pH 7.9, 10 mM KCl, 1.5 mM MgCl_2_, 1 mM DTT, 20 µg/ml aprotinin and leupeptin) on ice. The supernatant was centrifuged at 3300 *g* for 10 min at 4°C, resuspended in 5 vols of hypotonic buffer and incubated on ice for 10 min with gentle stirring. Cells were broken by Dounce homogenization on ice and centrifuged at 3300 g for 15 min at 4°C. The supernatant was centrifuged at 7500 g for 20 min at 4°C and then ultracentrifuged at 100, 0001 *g* for 4.5 h at 4°C. The supernatant was passed through a glass wool filter and then through a 0.45 µm filter. Samples were concentrated using Vivaspin concentrator (Amersham) and ∼3.8 mg of 60S HSE was used in pull down assays. Pellets containing HIV-1 vector were resuspended into 200 µl of binding buffer. To disrupt virus particles, 160 µl purified viral stock was sonicated for 6 sec. at 40 output level in an Ultrasonicator Process machine and 50 µl were used in pull down assays.

### Pull down assays

Mutant tRNA clones were obtained by PCR using mutagenic primers (see Table S1 and Table S2), generated by T7 polymerase and purified as previously described [15].

Pull-down assays were performed with ∼6.5 µg GST-Tnp3 or GST-Xpo-t, 3 µg tRNAs, 14 µg RanQ69L-GTP in a final volume of 30 µl binding buffer (50 mM Hepes pH 7.3, 200 mM NaCl, 2 mM Mg(Ac)_2_ and 10 µM GTP). The mix was incubated at 4°C for 30 mins with gentle mixing then 20 µl equilibrated Glutathione-sepharose 4B beads (GE Healthcare) were added and samples incubated for a further 2 h at 4°C with continuous rotation. Samples were washed with binding buffer (1 ml×4 washes), mixed with 30 µl 5x SDS-sample buffer and boiled for 10 mins, resolved by 10% SDS-PAGE and visualized by silver staining following the manufacturer's instructions (Silver Staining Plus, BioRad). ImageJ 1.42q (NIH) was used to quantify the intensity and the total area of the bands in silver stained gels following thresholding to optimize linearity of the signal. Values of the pulled down bands were normalized by the input bands in each sample.

### Export assays

Import assays were performed with fluorescently-labelled tRNAs as previously described [15], [77] except that incubation of permeabilized cells with tRNAs and the energy regenerating system was at 37°C for 10 mins. Samples were washed 3 times in import buffer (20 mM HEPES pH 7.3, 110 mM KAc, 5 mM Mg(Ac)_2_, 0.5 mM EGTA, 250 mM sucrose) on ice then the export mix was added (Tnp3 to 1 µM final concentration and 1x energy mix and 1xRan mix in 30 µl import buffer) and samples incubated at 37°C for 10 mins. Following three washes in import buffer, samples were fixed in 2% paraformaldehyde in import buffer for 5 mins on ice, washed twice in import buffer and analyzed by confocal microscopy. Quantitative analysis of fluorescent signal in the nuclei was performed on confocal images by MetaMorph software version 4.5r4 (Universal Imaging Corp., Molecular Devices) as previously described [15].

### Western blot

Rabbit polyclonal anti-importin 7 antibodies were previously described [77], monoclonal antibody 3152C2a against Tnp3 was purchased from Abcam (Cambridge, UK) and used 1/500 dilution in TBST-T. Anti-p24/p55 monoclonal antibodies EH12E1 and 3D3 were obtained from the AIDS repository reagent programme EVA centre for AIDS reagents, UK, mixed and used at 1/300 dilution. Anti-β-actin monoclonal antibody (AC-40, Sigma-Aldrich) was used at a 1/10,000 dilution. Anti-rabbit and anti-mouse IgG HRP-conjugated antibodies were purchased from Jackson Laboratories (Bar Harbor, MN) and from Sigma respectively. After SDS PAGE, the proteins were transferred overnight to a PVDF membrane (Bio-Rad, Hercules, CA) in transfer buffer pH 8.4 containing 0.01% SDS and 5% methanol and probed with the primary antibodies for 1 h at room temperature. HRP-conjugated secondary antibodies were used diluted 1/3,000 in 10% non-fat milk. Chemiluminescence (ECL, Amersham) was used to develop the blots as described by the manufacturer. Autoradiography films were exposed for different periods of time to ensure linearity of the signal.

### Cell fractionation and detection of viral DNA

Approximately 10^6^ HeLa cells were plated onto 10 cm plates. The next day cells were infected at an MOI of 0.2–0.5 for 22–24 hours, trypsinized and fractionation was performed in the presence of NP-40 as previously described [26], [78]. For DNAse I digestion, 10 µl DNAse I beads (MoBiTec, Göttingen, Germany) were added to the fractionation buffer with 5 mM CaCl_2_ and incubated at 4°C for 5 mins with rotation, followed by washes as previously described. TaqMan qPCR was performed in an ABI Prism 7000 thermocycler as described [78]. For amplification of 2LTR circular DNA, the same conditions were used with primers 2LTRqPCRF: 5′-AACTAGAGATCCCTCAGACCCTTTT-3′ and 2LTRqPCRRC: 5′-CTTGTCTTCGTTGGGAGTGAATT-3′ and probe 5′-FAM-CTAGAGTTTTCCACACTGAC-0-TAMRA-3′ [26]. Standards were prepared by PCR amplification of DNA from acutely infected cells with primers 2LTRF 5′-GCCTCAATAAAGCTTGCCTGG-3′ and 2LTRRC 5′-TCCCAGGCTCAGATCTGGTCTAAC-3′. The amplification product was cloned into TOPO vector, amplified and confirmed by sequencing. Detection of cyclophilin A cDNA was as described [26]. Alu-LTR Taqman qPCR was carried out as previously described [41] using primers ALU-forward, AAC TAG GGA ACC CAC TGC TTA AG and LTR1-reverse, TGC TGG GAT TAC AGG CGT GAG (for first round amplification) and ALU-forward AAC TAG GGA ACC CAC TGC TTA AG, LTR2-reverse, TGC TAG AGA TTT TCC ACA CTG ACT, ALU-probe, FAMRA – TAG TGT GTG CCC GTC TGT TGT GTG AC – TAM (for second round Taqman qPCR).

## Acknowledgments

We thank Woan-Yuh Tarn for the Tnp3 expression plasmid, Dirk Görlich for the Xpo-t expression plasmid, Michael Emerman, Adrian Thrasher, Didier Trono, and Masahiro Yamashita for the HIV-1 plasmids, Apsara Kandanearatchi for primary human macrophages, Ian Anderson and Luciano Vozzolo for assistance with experiments, Peter Cherepanov and Stephen Hare for helpful discussions, Greg Towers and members of his lab for reagents, Mahad Noursadeghi for help with image analysis.

## Supporting Information

Figure S1 Tnp3 facilitates integration of HIV-1 in CD4+ T-cells. (A) Jurkat cells were transduced with an MLV-based vector delivering an shRNA expression construct targeting human Tnp3 mRNA. Cells were normalised by cell number and knockdown of Tnp3 was examined by Western blotting. (B) HIV_GFP_ vector was used to transduce Tnp3-KD and wild type (WT) Jurkat cells. The percentage of GFP-positive cells was determined by flow cytometry. Average values ± SD of three independent experiments are shown. Total DNA was extracted from infected cells at the indicated time points and the amount of total linear viral DNA (C) and 2LTR circular DNA (D) copies per 100 ng total DNA were determined by Taqman qPCR. (E) Diagram showing ratio of total linear viral DNA to 2LTR circles. (F) The amount of integrated provirus copies/100 ng total DNA in Tnp3-KD and WT Jurkat cells was determined 10 days post infection by Alu-LTR Taqman qPCR. Average values ± SD of three independent experiments are shown.
(JPG)

Figure S2 Nuclear viral DNA is protected from DNAse I digestion. (A) Tnp3 KD cells were transduced at MOI of 0.5 with HIV-1_GFP_ and fractionated 24 h later in the presence of DNAse I beads. Viral DNA in each fraction was measured by Taqman qPCR. Average ± SD of triplicate determinations are shown, representative of two independent experiments. (B) HIV-1_GFP_ plasmid DNA was incubated in parallel with the same amount of DNAse I beads used for the cell fractionation, re-purified and subjected to Taqman qPCR.
(JPG)

Figure S3 Infection with VSV-G pseudotyped HIV LAIΔenv is impaired in Tnp3 KD HeLa cells. (A) Tnp3 KD and control (scramble) cells were infected with two doses of HIV LAIΔenv and analyzed by FACS 24 h and 48 h post-infection. Data are expressed as average percentage of infection relative to control (scramble siRNA) ± average deviation of two independent experiments performed in duplicate. (B) Total viral DNA and (C) 2LTRs circular viral DNA were quantified in Tnp3 KD and control cells by Taqman qPCR 24 h and 48 h post-infection. Average values ± average deviation of two independent experiments are shown, which were each performed in duplicate.
(JPG)

Figure S4 The viral envelope does not significantly influence susceptibility to Tnp3 KD. Polyclonal populations of TZM-bl cells stably expressing a control shRNA or a shRNA targeting Tnp3 were transduced at an MOI of 0.03 with HIV-1_GFP_ bearing the VSV-G or gp120 envelopes and analyzed by flow cytometry 48 hours later. Average values ± SD of three independent experiments are shown.
(JPG)

Figure S5 HIV-1 capsid is detected in the nucleus at 16 hours post-infection but the mutant N74D capsid remains cytoplasmic. (A) HeLa cells stably expressing a scrambled shRNA control were seeded onto glass coverslips and either left untreated (lower panel), or infected with WT virus (upper panel) or N74D mutant virus (middle panel) at an MOI of 5 for 16 h at 37°C. The cells were then washed to remove virus, fixed with formaldehyde, permeabilized with methanol and stained with mouse monoclonal antibody specific for HIV-1 Gag p24/p55 (clone EH12E1 donated by Dr. R.B. Ferns and Dr. R.S Tedder and obtained from the Center for AIDS Reagents, National Institute of Biological Standards and Control, UK) followed by an anti-mouse IgG1-Alexa543 conjugated secondary antibody (Invitrogen). Coverslips were mounted with ProLong Gold antifade containing DAPI (Invitrogen) and visualized using a Leica SP2 confocal microscope. Image processing was performed using Metamorph v7 and Photoshop. Images are single, /xy/ sections taken through the middle of the cell and the right hand panel shows a higher magnification image of the cells indicated with arrows. Scale bars are 5 µm. (B) Pull down assay with GST-Tnp3 in the presence or absence of RanQ69L-GTP and purified HIV-1 vector bearing the N74D mutation in CA. Viral tRNAs were detected by silver staining and p24 CA (N74D) was detected by Western blot. ImageJ quantification of pull down assays is shown in the bottom panels. Values are expressed as ratio of input versus recovered tRNAs.
(JPG)

Table S1 Sequences of the tRNA mutants used in pull downs and export assays. The complete sequences of the tRNAs generated by T7 polymerase are shown. Changes to the original G2 template are highlighted in red and underlined.
(DOC)

Table S2 Sequences of the primers used to generate the tRNA T7 template by PCR. The T7 promoter sequence is highlighted in blue. Mutants m20 and m22 were generated in two separate rounds of amplification. The forward primer sequences for the first amplification round (1^st^) are given, the second amplification round was primed with the G2 forward primer.
(DOC)
