# Supplementary figures and images for: Transportin 3 Promotes a Nuclear Maturation Step Required for Efficient HIV-1 Integration
Source: PLoS Pathog. 2011 Aug 25;7(8):e1002194. doi: 10.1371/journal.ppat.1002194 (PMC3161976; doi:10.1371/journal.ppat.1002194)

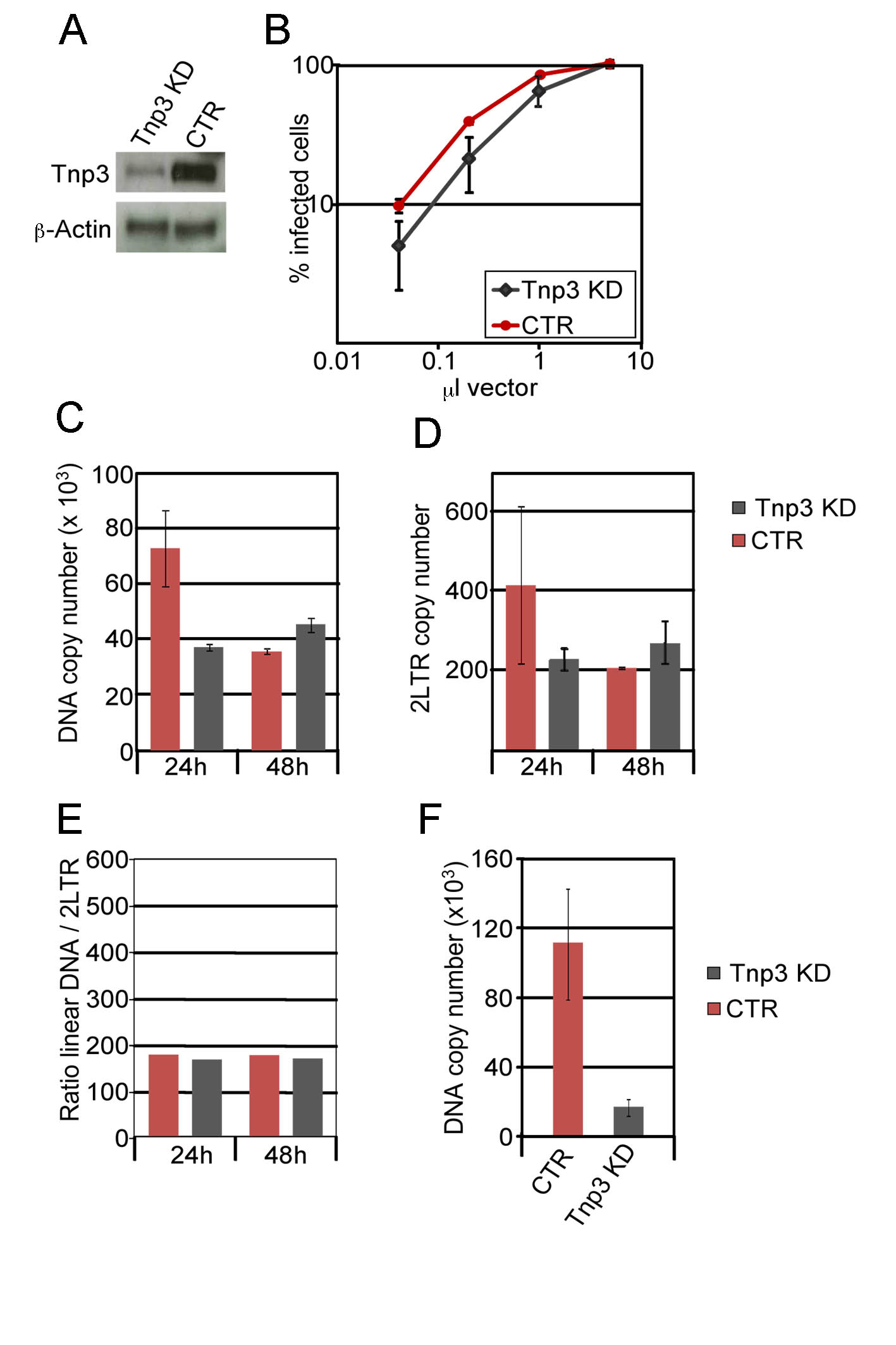

Supplement: Figure S1 — Tnp3 facilitates integration of HIV-1 in CD4+ T-cells. (A) Jurkat cells were transduced with an MLV-based vector delivering an shRNA expression construct targeting human Tnp3 mRNA. Cells were normalised by cell number and knockdown of Tnp3 was examined by Western blotting. (B) HIVGFP vector was used to transduce Tnp3-KD and wild type (WT) Jurkat cells. The percentage of GFP-positive cells was determined by flow cytometry. Average values ± SD of three independent experiments are shown. Total DNA was extracted from infected cells at the indicated time points and the amount of total linear viral DNA (C) and 2LTR circular DNA (D) copies per 100 ng total DNA were determined by Taqman qPCR. (E) Diagram showing ratio of total linear viral DNA to 2LTR circles. (F) The amount of integrated provirus copies/100 ng total DNA in Tnp3-KD and WT Jurkat cells was determined 10 days post infection by Alu-LTR Taqman qPCR. Average values ± SD of three independent experiments are shown. (JPG) [file ppat.1002194.s001.jpg]

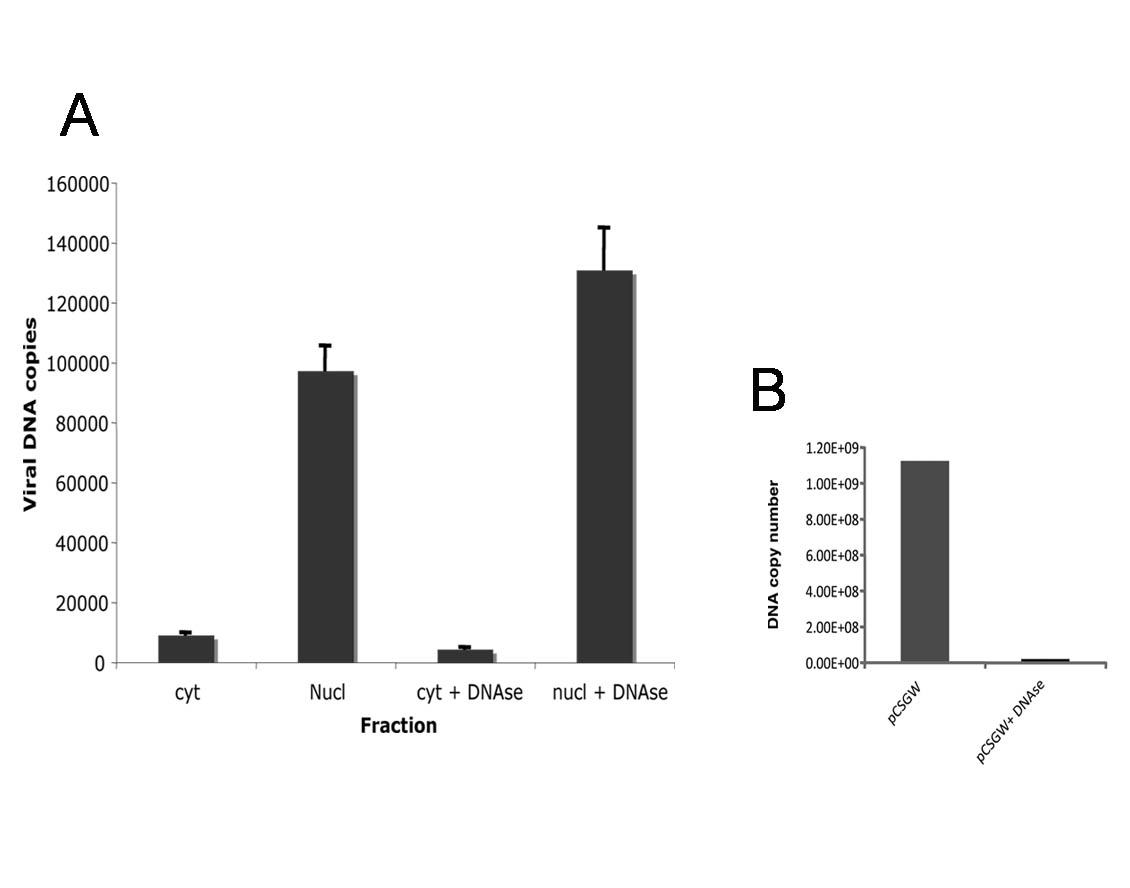

Supplement: Figure S2 — Nuclear viral DNA is protected from DNAse I digestion. (A) Tnp3 KD cells were transduced at MOI of 0.5 with HIV-1GFP and fractionated 24 h later in the presence of DNAse I beads. Viral DNA in each fraction was measured by Taqman qPCR. Average ± SD of triplicate determinations are shown, representative of two independent experiments. (B) HIV-1GFP plasmid DNA was incubated in parallel with the same amount of DNAse I beads used for the cell fractionation, re-purified and subjected to Taqman qPCR. (JPG) [file ppat.1002194.s002.jpg]

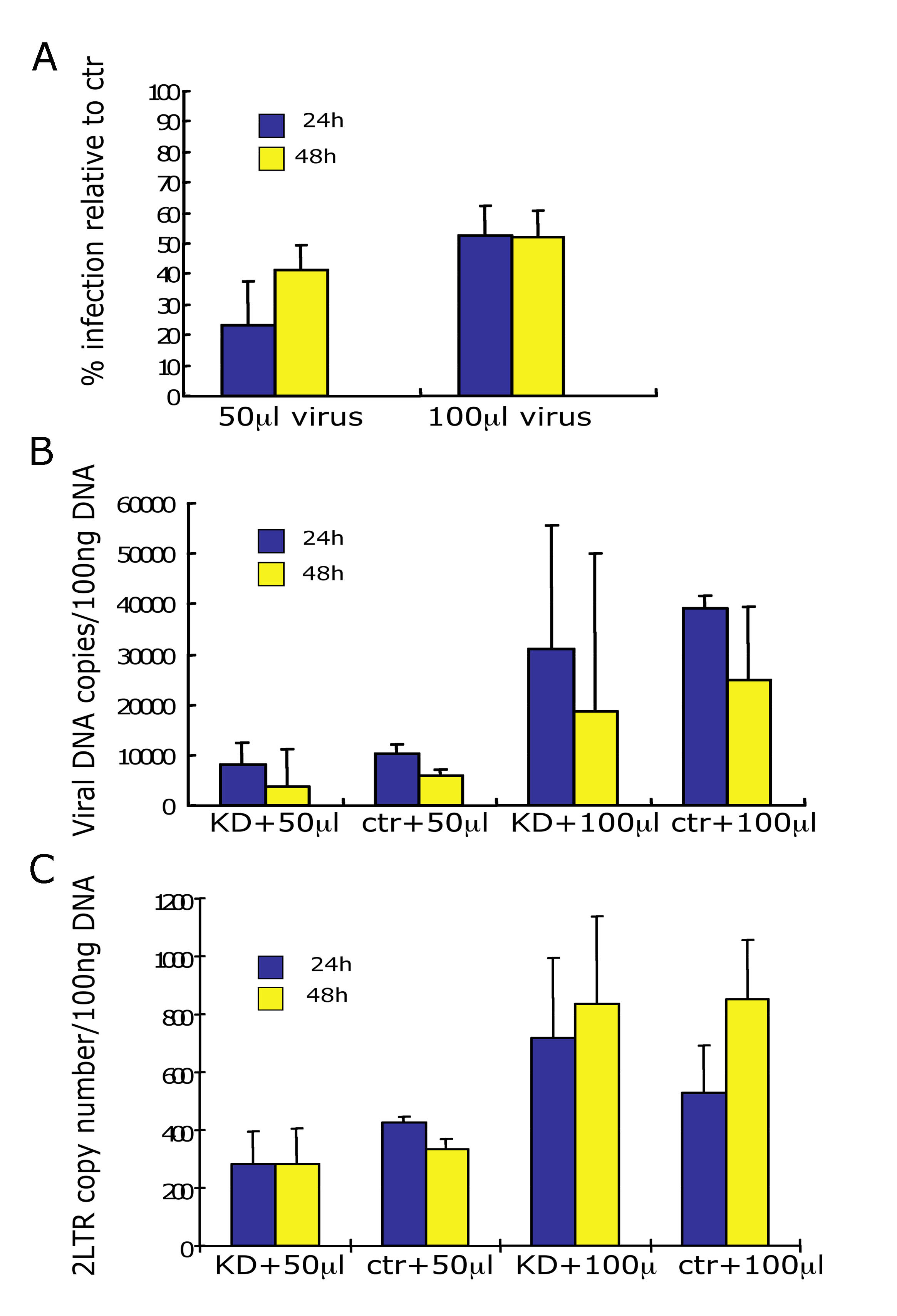

Supplement: Figure S3 — Infection with VSV-G pseudotyped HIV LAIΔenv is impaired in Tnp3 KD HeLa cells. (A) Tnp3 KD and control (scramble) cells were infected with two doses of HIV LAIΔenv and analyzed by FACS 24 h and 48 h post-infection. Data are expressed as average percentage of infection relative to control (scramble siRNA) ± average deviation of two independent experiments performed in duplicate. (B) Total viral DNA and (C) 2LTRs circular viral DNA were quantified in Tnp3 KD and control cells by Taqman qPCR 24 h and 48 h post-infection. Average values ± average deviation of two independent experiments are shown, which were each performed in duplicate. (JPG) [file ppat.1002194.s003.jpg]

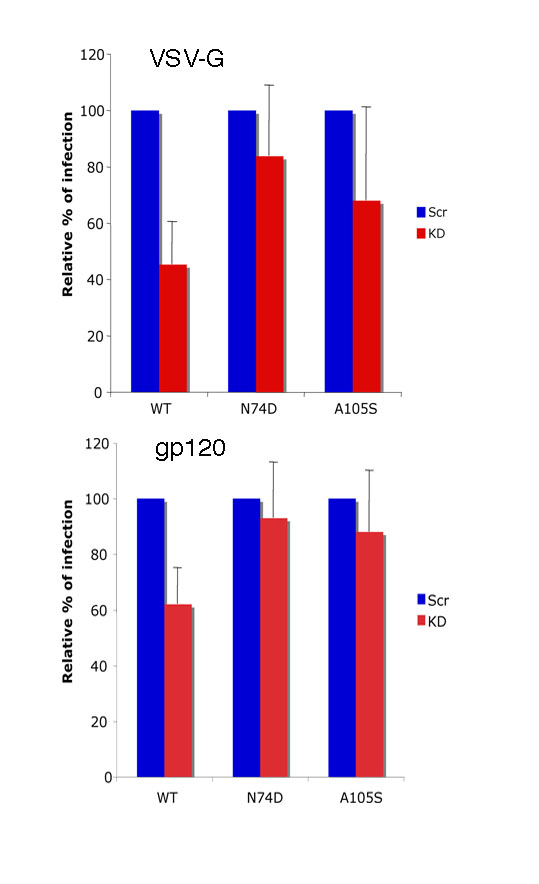

Supplement: Figure S4 — The viral envelope does not significantly influence susceptibility to Tnp3 KD. Polyclonal populations of TZM-bl cells stably expressing a control shRNA or a shRNA targeting Tnp3 were transduced at an MOI of 0.03 with HIV-1GFP bearing the VSV-G or gp120 envelopes and analyzed by flow cytometry 48 hours later. Average values ± SD of three independent experiments are shown. (JPG) [file ppat.1002194.s004.jpg]

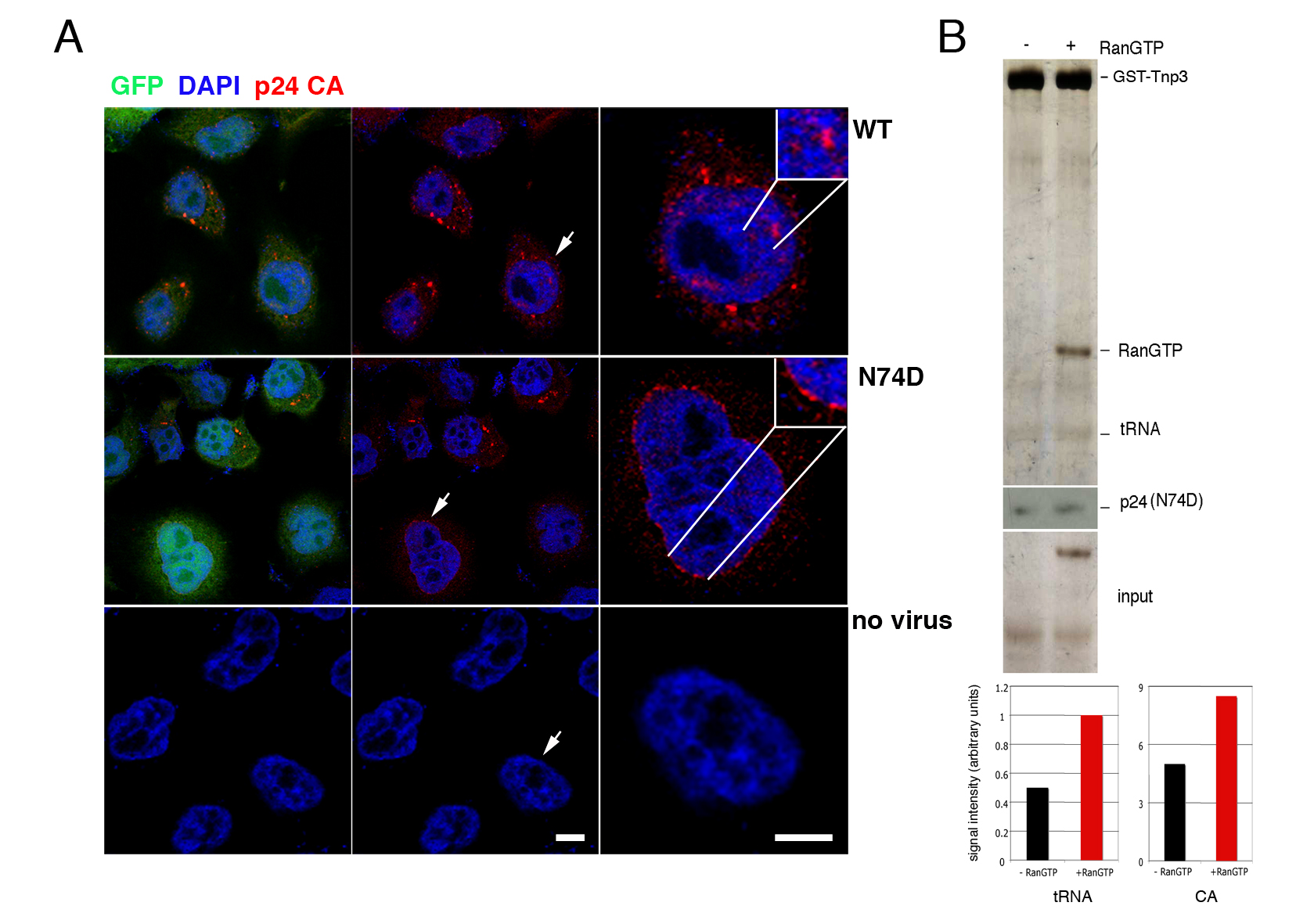

Supplement: Figure S5 — HIV-1 capsid is detected in the nucleus at 16 hours post-infection but the mutant N74D capsid remains cytoplasmic. (A) HeLa cells stably expressing a scrambled shRNA control were seeded onto glass coverslips and either left untreated (lower panel), or infected with WT virus (upper panel) or N74D mutant virus (middle panel) at an MOI of 5 for 16 h at 37°C. The cells were then washed to remove virus, fixed with formaldehyde, permeabilized with methanol and stained with mouse monoclonal antibody specific for HIV-1 Gag p24/p55 (clone EH12E1 donated by Dr. R.B. Ferns and Dr. R.S Tedder and obtained from the Center for AIDS Reagents, National Institute of Biological Standards and Control, UK) followed by an anti-mouse IgG1-Alexa543 conjugated secondary antibody (Invitrogen). Coverslips were mounted with ProLong Gold antifade containing DAPI (Invitrogen) and visualized using a Leica SP2 confocal microscope. Image processing was performed using Metamorph v7 and Photoshop. Images are single, /xy/ sections taken through the middle of the cell and the right hand panel shows a higher magnification image of the cells indicated with arrows. Scale bars are 5 µm. (B) Pull down assay with GST-Tnp3 in the presence or absence of RanQ69L-GTP and purified HIV-1 vector bearing the N74D mutation in CA. Viral tRNAs were detected by silver staining and p24 CA (N74D) was detected by Western blot. ImageJ quantification of pull down assays is shown in the bottom panels. Values are expressed as ratio of input versus recovered tRNAs. (JPG) [file ppat.1002194.s005.jpg]
