# Supplementary material for: Transportin 3 Promotes a Nuclear Maturation Step Required for Efficient HIV-1 Integration
Source: PLoS Pathog. 2011 Aug 25;7(8):e1002194. doi: 10.1371/journal.ppat.1002194 (PMC3161976; doi:10.1371/journal.ppat.1002194)
Supplement: Table S1 — Sequences of the tRNA mutants used in pull downs and export assays. The complete sequences of the tRNAs generated by T7 polymerase are shown. Changes to the original G2 template are highlighted in red and underlined. (DOC) [file ppat.1002194.s006.doc]

| Mutant number | mutation | Sequence (5’-3’) |
| --- | --- | --- |
| G2 (Lys^1,2^ no CCA 3’ end) | - | GCCCGGCTAGCTCAGTCGGTAGAGCATGAGACTCTTAATCTCAGGGTCGTGGGTTCGAGCCCCACGTTGGGCG |
| m2 | +TTT at 3’ end | GCCCGGCTAGCTCAGTCGGTAGAGCATGAGACTCTTAATCTCAGGGTCGTGGGTTCGAGCCCCACGTTGGGCGTTT |
| m2a | +CCA at 3’end | GCCCGGCTAGCTCAGTCGGTAGAGCATGAGACTCTTAATCTCAGGGTCGTGGGTTCGAGCCCCACGTTGGGCGCCA |
| m5 | U55A | GCCCGGCTAGCTCAGTCGGTAGAGCATGAGACTCTTAATCTCAGGGTCGTGGGTACGAGCCCCACGTTGGGCG |
| m6 | G18U/U55G | GCCCGGCTAGCTCAGTCUGTAGAGCATGAGACTCTTAATCTCAGGGTCGTGGGTGCGAGCCCCACGTTGGGCG |
| m10 | U54A | GCCCGGCTAGCTCAGTCGGTAGAGCATGAGACTCTTAATCTCAGGGTCGTGGGATCGAGCCCCACGTTGGGCG**-** |
| m20 | Lys  Glu | GCCCGGCTAGCTCAGTCGGTAGAGCATGAGACTCTCAATCTCAGGGTCGTGGGTTCGAGCCCCACGTTGGGCG |
| m22 | Lys  Asp | GCCCGGCTAGCTCAGTCGGTAGAGCATGAGACTGTCAATCTCAGGGTCGTGGGTTCGAGCCCCACGTTGGGCG |

**Supporting Table 1.** Sequences of the tRNA mutants.
