# Supplementary material for: Transportin 3 Promotes a Nuclear Maturation Step Required for Efficient HIV-1 Integration
Source: PLoS Pathog. 2011 Aug 25;7(8):e1002194. doi: 10.1371/journal.ppat.1002194 (PMC3161976; doi:10.1371/journal.ppat.1002194)
Supplement: Table S2 — Sequences of the primers used to generate the tRNA T7 template by PCR. The T7 promoter sequence is highlighted in blue. Mutants m20 and m22 were generated in two separate rounds of amplification. The forward primer sequences for the first amplification round (1st) are given, the second amplification round was primed with the G2 forward primer. (DOC) [file ppat.1002194.s007.doc]

| Mutant number | Forward primer | Reverse primer |
| --- | --- | --- |
| G2 | *AGTTAATACGACTCACTATAGG*GCCCGGCTAGCTCAGTCGGTAG | CGCCCAACGTGGGGCTCGAAC |
| m2 | *AGTTAATACGACTCACTATAGG*GCCCGGCTAGCTCAGTCGGTAG | AAACGCCCAACGTGGGGCTCGAAC |
| m2a | *AGTTAATACGACTCACTATAGG*GCCCGGCTAGCTCAGTCGGTAG | TGGCGCCCAACGTGGGGCTCGAAC |
| m5 | *AGTTAATACGACTCACTATAGG*GCCCGGCTAGCTCAGTCGGTAG | CGCCCAACGTGGGGCTCGTACCCA |
| m6 | *AGTTAATACGACTCACTATAGG*GCCCGGCTAGCTCAGTCTGTAG | CGCCCAACGTGGGGCTCGCACCCA |
| m10 | *AGTTAATACGACTCACTATAGG*GCCCGGCTAGCTCAGTCGGTAG | CGCCCAACGTGGGGCTCGATCCCA |
| m20 | CCCTAGCTCAGTCGGTAGAGCATGAGACTCTCAATCTCA (**1^st^**) | CGCCCAACGTGGGGCTCGAAC |
| m22 | CCCTAGCTCAGTCGGTAGAGCATGAGACTGTCAATCTCA (**1^st^**) | CGCCCAACGTGGGGCTCGAAC |

**Supporting Table 2**
